# Identification and characterization of survival-dependent genes in esophageal cancer via the DepMap database: unraveling their association with immune infiltration

**DOI:** 10.1007/s12672-025-02942-0

**Published:** 2025-06-22

**Authors:** Xiangrong Yao, Junyan He, Wentao Xiao, Limou Chen, Fangzhu Xiao

**Affiliations:** 1 School of Public Health, University of South China, Hengyang City, Hunan Province China; 2 Department of Oncology Radiotherapy, The First Affiliated Hospital, Hengyang Medical School, University of South China, Hengyang City, Hunan Province China; 3 Department of Radiation Oncology, Medical School of Nantong university, Affiliated Hospital of Nantong University, Nantong university, Nantong city, Jiangsu Province China; 4 School of Public Health, Xiangnan University, ChenZhou City, Hunan Province China

**Keywords:** Esophageal Cancer, Biomarkers, Prognostic model, Immune infiltration

## Abstract

**Background:**

Esophageal cancer ranks as the 11th most diagnosed cancer worldwide and the 7th leading cause of cancer-related deaths, mainly due to late-stage diagnosis. Identifying novel biomarkers is essential for enhancing prognostic evaluations and targeting patients for immunotherapy.

**Methods:**

We used the DepMap database to identify survival-dependent genes in esophageal carcinoma cells. A prognostic model was developed using univariate and multivariate Cox regression and LASSO, validated with the GEO dataset. WGCNA and GSEA analyses were conducted to explore mechanisms, alongside ESTIMATE and ssGSEA for prognosis.

**Results:**

We constructed a novel four-gene prognostic signature (CPSF6, IGBP1, MTG2, TCP1) based on SDG expression and survival data. This signature stratified esophageal cancer patients into high- and low-risk groups with significantly different survival, with the high-risk group showing shorter survival. WGCNA and GSEA analyses linked prognosis to immune pathways, including interferon-γ response and IL6-JAK-STAT3 signaling. ssGSEA revealed reduced infiltration of 19 immune cell types in high-risk patients, and ESTIMATE analysis confirmed the association between immune infiltration and poor prognosis.

**Conclusion:**

This study establishes a four-gene survival signature for esophageal cancer that distinguishes high-risk from low-risk populations, providing novel prognostic indicators. Immune response pathways were downregulated in high-risk patients, offering potential targets for understanding esophageal cancer mechanisms and developing immunotherapeutic strategies.

**Supplementary Information:**

The online version contains supplementary material available at 10.1007/s12672-025-02942-0.

## Introduction

Esophageal cancer is the 11th most commonly diagnosed cancer worldwide and the 7th leading cause of cancer-related mortality, with approximately 511,000 new cases and 445,000 deaths reported in 2022. This highlights a significant public health concern. A disparity exists between the sexes, with incidence and mortality rates varying by a factor of 2 to 3. In transitional and developing countries, incidence and mortality rates are slightly higher in males than in females; however, the opposite trend is observed in some populations of females [1]. In the field of esophageal cancer (EC), China accounts for more than 50% of global cases, which is particularly concerning as it disproportionately affects disadvantaged groups who often present with more severe conditions [2].

Esophageal cancer primarily presents as two histological subtypes: esophageal squamous cell carcinoma (ESCC) and adenocarcinoma (EAC). These two subtypes are associated with distinct risk factors and incidence trends. While the incidence of ESCC is declining in many regions worldwide, in contrast, the incidence of EAC has dramatically increased in developed countries over the past forty years [3].

Esophageal squamous cell carcinoma (ESCC) is linked to various dietary factors, including alcohol, tobacco, and opiate use, as well as environmental pollution, the consumption of hot beverages, nutritional deficiencies, exposure to pickled foods, and elevated levels of nitrosamines [4, 5]. The risk factors for esophageal adenocarcinoma (EAC) comprise gastroesophageal reflux disease, central obesity, and smoking, which are critical for understanding the etiology of this cancer type [6]. EAC is primarily seen in males, with a male-to-female ratio reaching up to 9:1 [7].

Treating esophageal squamous cell carcinoma (ESCC) is widely acknowledged as challenging due to its high rates of complications and mortality, a poor prognosis, and a significant risk of recurrence. These factors complicate treatment options and contribute to the overall difficulty in managing this disease [8, 9].The prognosis for esophageal squamous cell carcinoma (ESCC) is extremely poor, characterized by a high mortality rate. This grim outlook is partly attributed to the fact that ESCC is often diagnosed at advanced stages, primarily through enhanced chest computed tomography (CT) and endoscopy. Early detection remains a significant challenge, which further complicates treatment options [10]. Patients with esophageal adenocarcinoma (EAC) share characteristics with those of liver, lung, and pancreatic cancers. The 5-year overall survival rate for EAC is a mere 16%, with a median survival time of less than one year. This stark statistic highlights the severity of the disease. EAC is frequently diagnosed at advanced stages, with the majority of patients presenting with T3 or T4 disease. Consequently, this late-stage diagnosis contributes to the high mortality rate, as there are limited therapeutic options available for curing patients with advanced disease [11, 12].

In summary, esophageal squamous cell carcinoma and esophageal adenocarcinoma encounter significant challenges, such as difficulties in early diagnosis, rapid disease progression, and limited effective treatment options in advanced stages, leading to poor patient prognosis and high mortality rates.These challenges threaten patient health and life and highlight the urgent need for improvements in current diagnostic and therapeutic systems for esophageal cancer. This situation necessitates innovative research to develop more effective diagnostic and treatment strategies.

A review of previous studies suggests that single-gene biomarkers for esophageal cancer are negatively impacted by tumor heterogeneity and individual physiological variations, leading to decreased stability and reliability. This limitation has driven researchers to focus on multi-gene collaborative studies, as the synergistic analysis of multiple genes offers potential for addressing the shortcomings of single-gene approaches in capturing the complex biological behavior of tumors. By examining the interactions and expression patterns among genes, a more stable and predictive biomarker system can be developed, offering a more reliable foundation for clinical diagnosis and treatment. Furthermore, while immunotherapy has advanced in cancer treatment, there is still a limited understanding in academia of how the tumor microenvironment affects the progression of esophageal cancer, notably the critical role of immune suppression in its occurrence and development. This significantly hampers the effectiveness of immunotherapy in clinical applications for esophageal cancer.

**Fig. 1:**
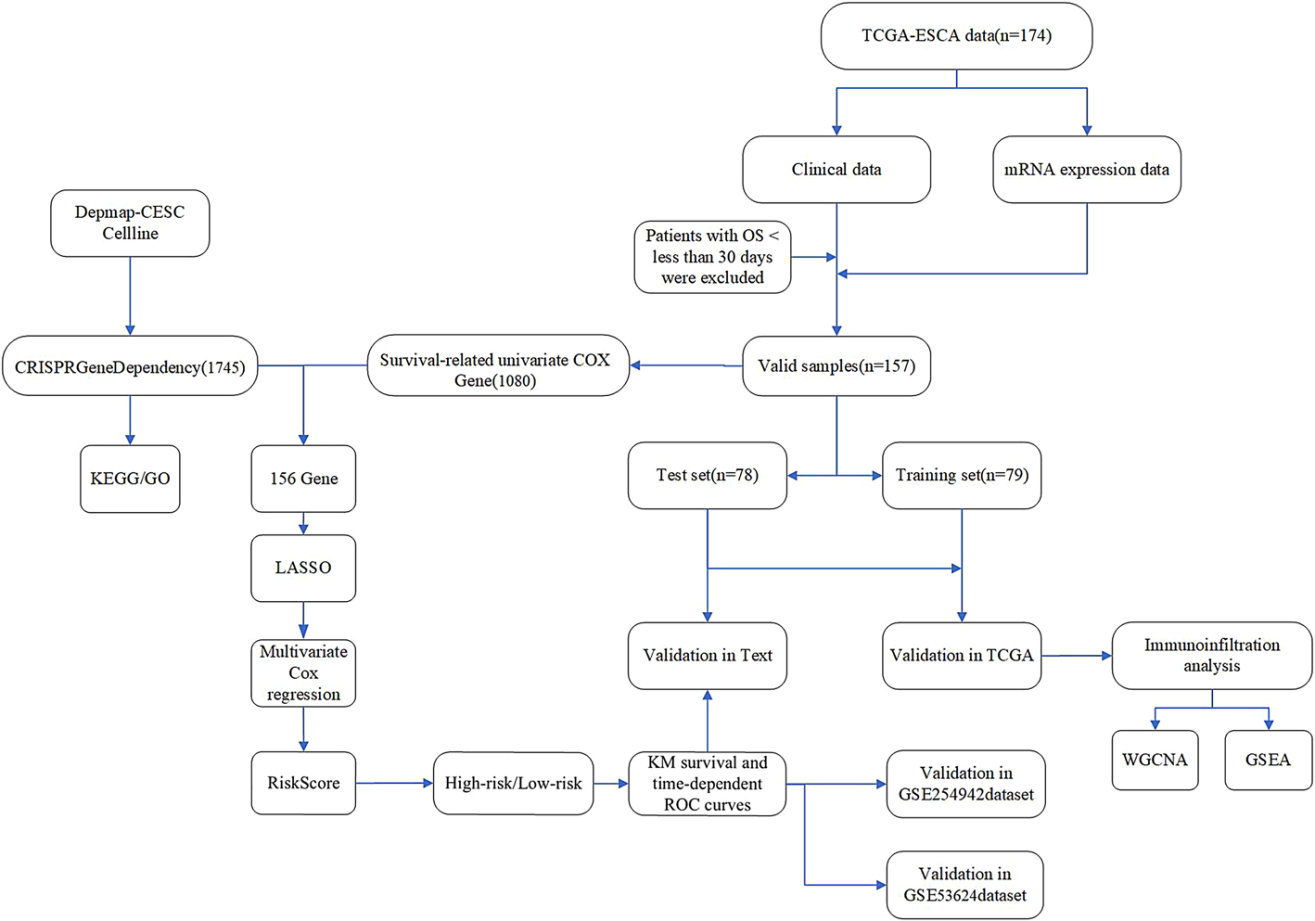
Flowchart of the methodological steps in our in-depth research

Accordingly, this study aims to break through existing bottlenecks by employing a whole-genome CRISPR-Cas9 knockout screening system to thoroughly dissect the key cancer dependencies in esophageal cancer. This study investigates the prognosis of esophageal cancer by selecting survival-dependent genes (SDGs) derived from cervical cancer cells in the DepMap database. A prognostic model was developed using univariate and multivariate Cox regression analyses combined with the LASSO algorithm, followed by validation using the GEO dataset. Gene modules were constructed using Weighted Gene Co-expression Network Analysis (WGCNA), and molecular mechanisms were analyzed through Gene Set Enrichment Analysis (GSEA).Furthermore, the tumor immune microenvironment was assessed using the ESTIMATE algorithm and single-sample Gene Set Enrichment Analysis (ssGSEA) to investigate its intrinsic relationship with esophageal cancer prognosis, as detailed in Fig. 1. This research not only holds promise for providing more accurate stratification tools and prognostic assessment metrics for esophageal cancer patients but also contributes to the identification of potential therapeutic targets and optimization of clinical treatment strategies.

## Materials and methods

### Identification of survival-dependent genes associated with esophageal cancer

By utilizing the Cancer Dependency Map (DepMap) database (https://depmap.org/portal/), we can access gene dependency data for a variety of cancer cell lines. The Chronos gene effect score is inferred to represent the probability of true dependency relationships, with an average value greater than 0.5 [13, 14] regarded as associated with survival.

The RNA sequencing (RNA-seq) transcriptomic expression data and the associated clinical information for patients with Esophageal cancer (EC) were obtained from the “TCGA-ESCA(n = 174)” project within The Cancer Genome Atlas (TCGA) database. To identify survival-dependent genes, univariate Cox, LASSO regression, and multivariate Cox analyses were performed. For validation, RNA-seq data and clinical information for patients were downloaded from the Gene Expression Omnibus (GEO) database (https://www.ncbi.nlm.nih.gov/geo/) from datasets GSE254942(*n* = 106) and GSE53624(*n* = 238), and all data processing was conducted using R (version 4.4.1).

### KEGG and GO function enrichment analysis

The potential biological functions of hub genes were examined using the “clusterProfiler” R package (version 4.12.6), which conducted enrichment analyses for the Kyoto Encyclopedia of Genes and Genomes (KEGG) and Gene Ontology (GO). In this analysis, an adjusted p-value of less than 0.05 was considered statistically significant.

### Development of a risk score model for survival-dependent genes

After excluding samples with a survival time of less than 30 days, we randomly divided 157 patients into training and testing sets at a 1:1 ratio to develop the optimal risk scoring model for survival-dependent genes. In the training set, we initially employed a univariate Cox regression method based on survival-dependent mRNAs to evaluate potential prognostic mRNAs. This approach allowed us to identify key mRNAs that significantly correlate with patient survival. Subsequently, the “GLMnet” R package was utilized to conduct Least Absolute Shrinkage and Selection Operator (LASSO) Cox regression analysis to select genes associated with esophageal cancer prognosis. After this selection process, a multivariate Cox regression method was employed for further optimization of the identified genes. The risk score for survival-dependent genes is computed using the following formula: Risk Score = (CPSF6 expression × 1.453) + (IGBP1 expression × 1.227) + (MTG2 expression × 1.074) + (TCP1 expression × 1.340). The coefficients corresponding to each gene are retained to three decimal places, specifically, CPSF6 coefficient is 1.453, IGBP1 coefficient is 1.227, MTG2 coefficient is 1.074, and TCP1 coefficient is 1.340.This formula enables the quantification of the combined effect of these genes on patient survival. Upon completion of the risk scoring model construction, patients were classified into high-risk and low-risk groups according to the median risk score. Kaplan-Meier survival analysis along with the log-rank test was employed to compare the survival differences between the two groups and assess the prognostic predictive performance of the model.

### Nomogram construction

By integrating survival-dependent risk features with clinical information from patients, a multivariate Cox regression analysis was conducted to identify independent factors affecting Esophageal cancer (EC) patients. To enhance the predictive power of our model, a nomogram was developed using the risk score and other clinical pathological characteristics. The calibration curve and C-index value of the nomogram were subsequently calculated to evaluate the correlation between the predicted probabilities and the actual incidence rates. In this analysis, a p-value of less than 0.05 was deemed statistically significant.

### WGCNA

In this study, we employed the “WGCNA” R package (version 1.73) to conduct Weighted Gene Co-expression Network Analysis. Initially, we retrieved gene expression data from esophageal cancer (ESCA) patients in The Cancer Genome Atlas (TCGA) database and categorized these patients into high-risk and low-risk groups according to their risk scores. This categorization allowed for a more detailed exploration of gene co-expression patterns associated with each risk group.

Subsequently, the average linkage hierarchical clustering method was employed to cluster the included samples for the identification of potential co-expression modules. To optimize the network construction, we selected a soft threshold parameter of β = 8 to construct a scale-free network. Using this approach, a total of 12 modules were identified. These modules may provide insights into the underlying biological processes related to esophageal cancer.

Finally, the Pearson correlation coefficient method was used to assess the correlation between the merged modules and the high-risk group, identifying several modules associated with the high-risk group of esophageal cancer. Notably, the black, yellow, magenta, pink, red, and green modules were found to be particularly significant. These findings suggest that these modules may play important roles in the progression of esophageal cancer.

### GSEA

Using the “clusterProfiler” R package (version 4.12.6) for Gene Set Enrichment Analysis (GSEA), each esophageal cancer patient was classified into high-risk and low-risk groups based on survival-dependent risk characteristics to identify relevant pathways. This classification allowed us to perform a comprehensive analysis of the biological pathways associated with each risk group. A p-value of less than 0.05 was deemed statistically significant.

### Immune profiling analysis

We compared immune scores, stromal scores, and ESTIMATE scores between the high-risk and low-risk groups using the “Estimate” R package. Following this comparison, we conducted single-sample gene set enrichment analysis (ssGSEA) using the “GSVA” R package to clearly illustrate the infiltration scores of 28 tumor-infiltrating immune cells in the high-risk and low-risk groups. This analysis provided insights into the immune landscape associated with different risk profiles.

## Results

### Identification of potential SDGs in esophageal cancer

**Fig. 2:**
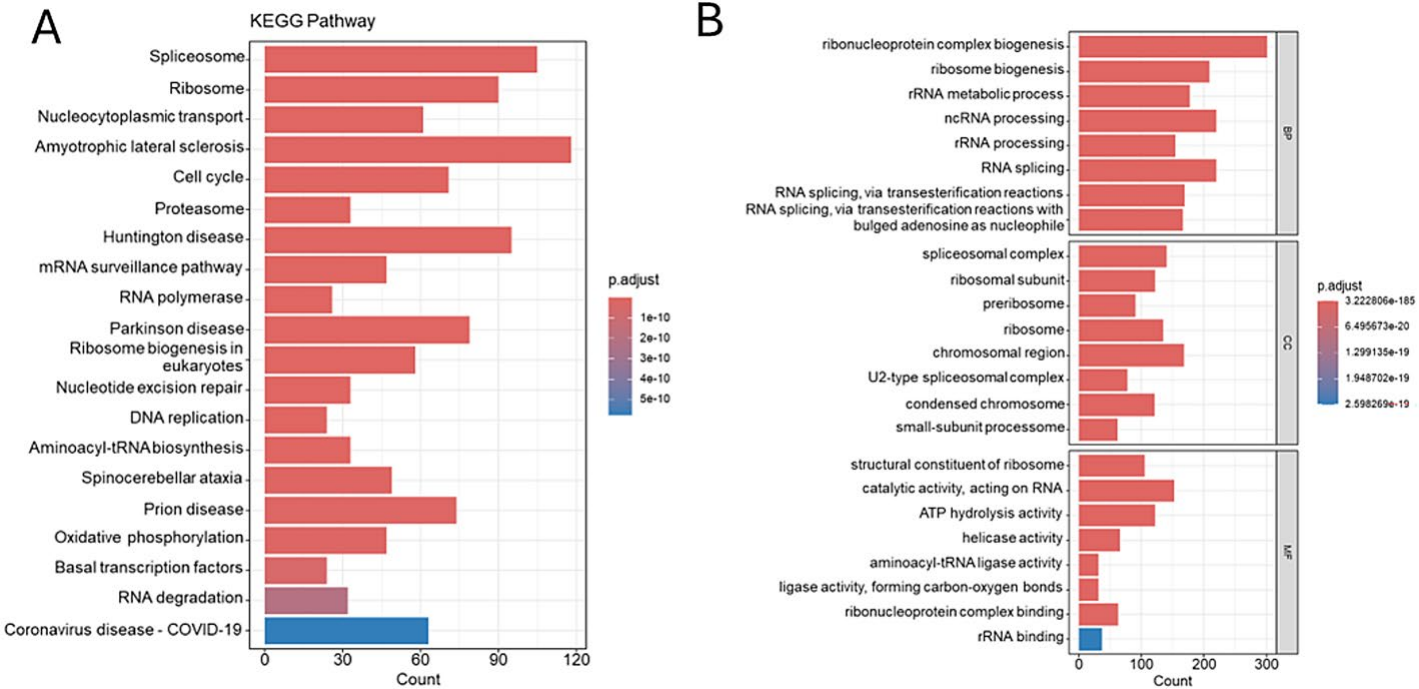
Identification of survival-dependent genes. (**A**) SDGs were gathered for KEGG enrichment analysis. (**B**) SDGs were gathered for GO enrichment analysis

Utilizing the DepMap Achilles project database, this research conducted an analysis of 31 ESCA cell lines, which included 7 ESCC cell lines and 24 EAC cell lines. Following a stringent selection process, 1,745 genes with an average dependency score greater than 0.5 were ultimately identified, regarded as potential survival dependency genes (SDGs). Subsequently, KEGG enrichment analysis (Fig. 2A) of these 1,745 genes demonstrated significant enrichment in biological processes including the cell cycle, ribosome biogenesis, proteasome function, DNA replication, RNA degradation, and oxidative phosphorylation.

Gene Ontology (GO) enrichment analysis (Fig. 2B) indicated that SDGs were primarily enriched in biological processes (BP) associated with ribonucleoprotein complex biogenesis and ribosome biogenesis. In terms of cellular components (CC), SDGs were predominantly enriched in spliceosomal complexes and ribosomes. They were primarily enriched in molecular functions (MF) related to ATP hydrolysis activity.

### Identification of Prognosis-Associated sdgs, development of prognostic features, and validation

**Fig. 3:**
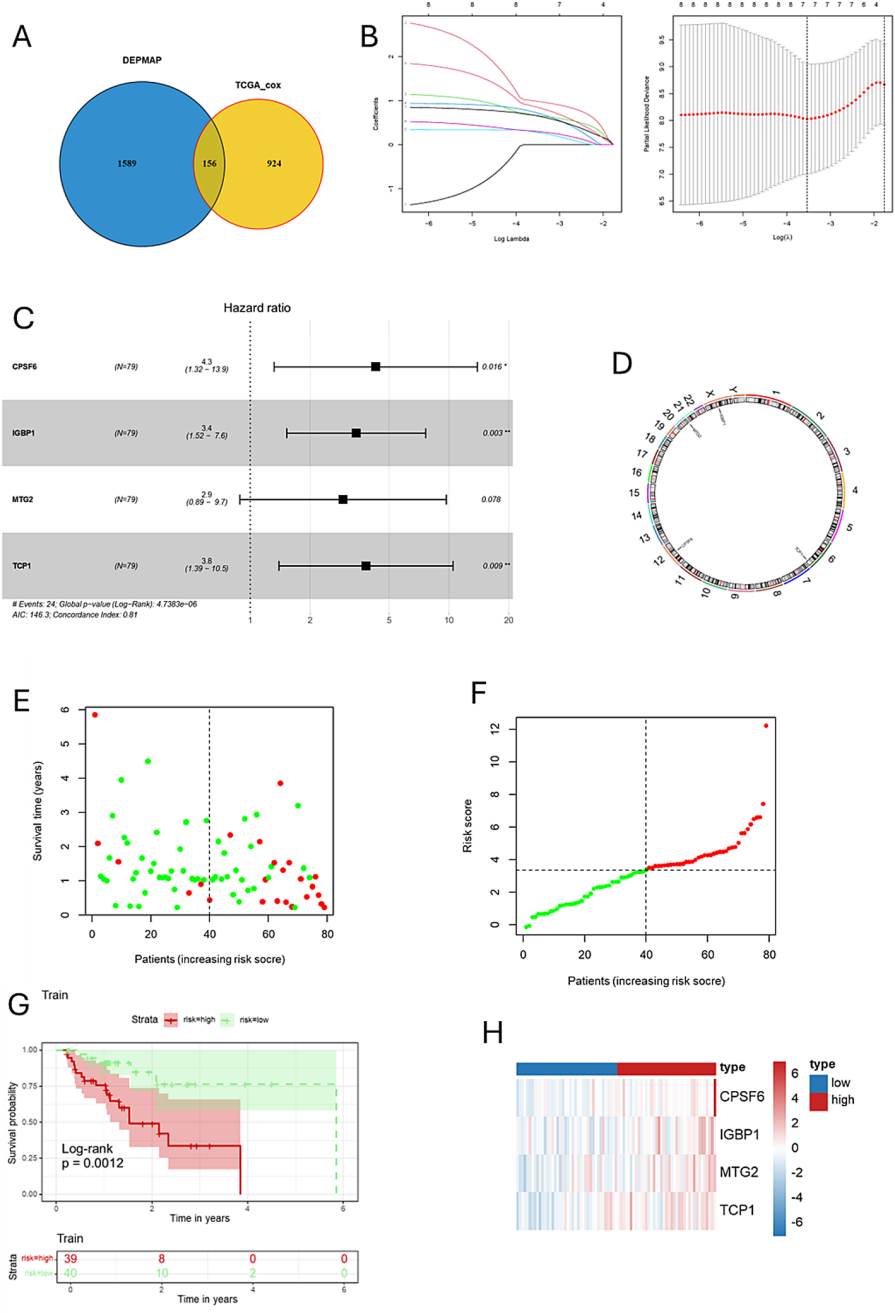
Construction and validation of an ESCA-related risk signature for prognostic assessment in the training set. (**A**) Gene intersection analysis of survival-dependent genes between TCGA and SDGs. (**B**) Utilizing the LASSO-Cox regression model, we calculated coefficients of potential biomarkers, ultimately identifying a set of proliferation-essential dependence genes (SDGs) that are significantly associated with ESCA prognosis. (**C**) Forest plot of prognostic proliferation-related genes in the training group. (**D**) The location of genes on chromosomes. (**E**, **F**). The risk scores and survival status distributions of patients within the train cohort were presented and analyzed. (**G**) Plot showing the Kaplan–Meier distribution for the train cohort. (**H**) Heatmap of the expression of these genes in the train group

**Fig. 4:**
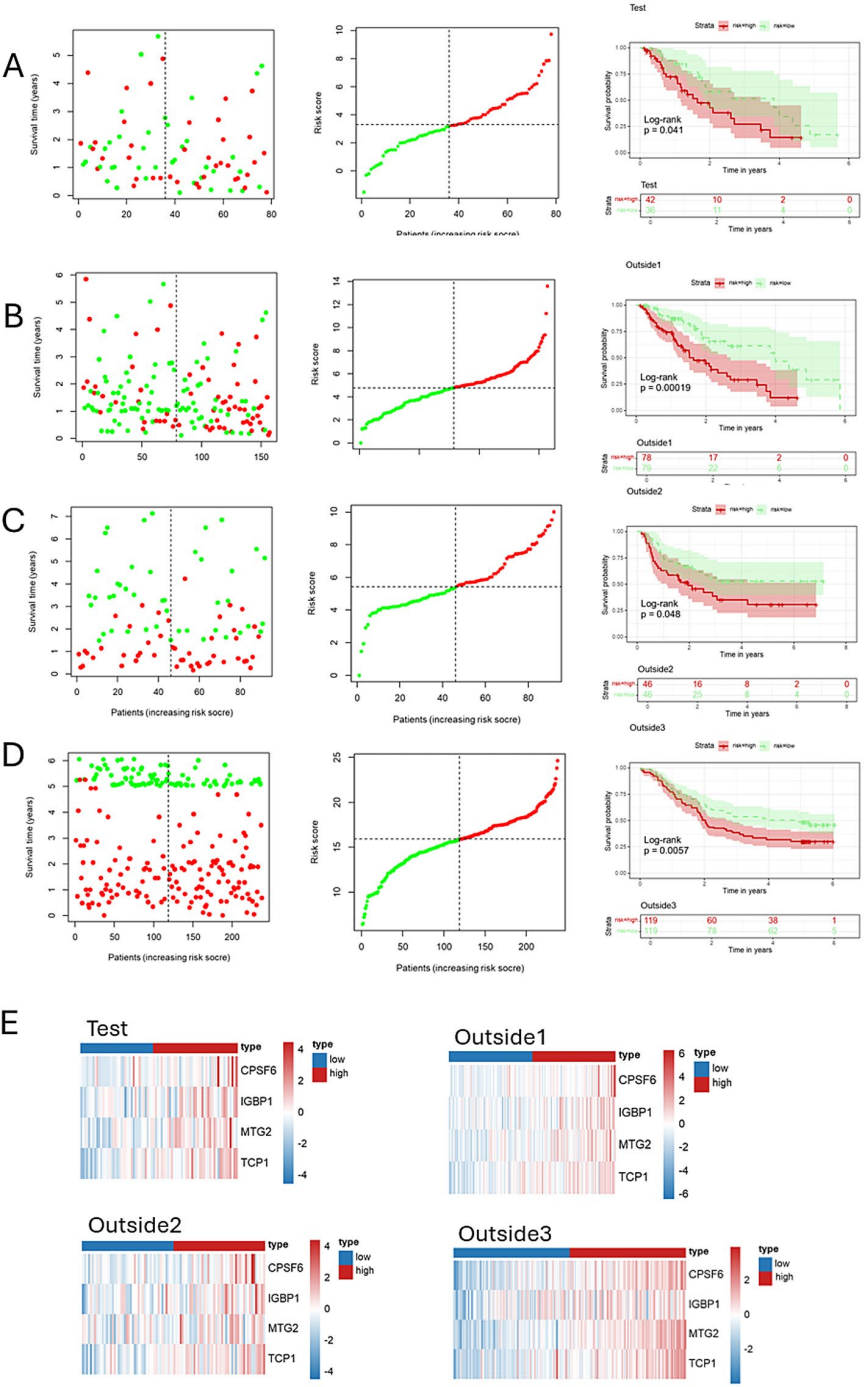
Results are presented for the Test cohort, the TCGA External Validation cohort, and the GSE254942 and GSE53624 External Validation cohorts. (**A**) Distribution of risk scores and survival status in the test cohort, along with a Kaplan-Meier plot illustrating the survival distribution. (**B**) Distribution of risk scores and survival status in the External Validation cohort TCGA, along with a Kaplan-Meier plot illustrating the survival distribution (**C**) Distribution of risk scores and survival status in the External Validation cohort GSE254942, along with a Kaplan-Meier plot illustrating the survival distribution. (**D)** Distribution of risk scores and survival status in the External Validation cohort GSE53624, along with a Kaplan-Meier plot illustrating the survival distribution (**E**) Heatmap Illustrating Gene Expression Levels in the Test Cohort, TCGA - ESCA Cohort, and Survival - Prediction - Oriented GSE254942 and GSE53624 Cohorts

After excluding samples with a survival time of less than 30 days, we randomly divided the TCGA-ESCA cohort (*n* = 157) into two groups: a training group of 79 patients and a testing group of 78 patients. From the 1,080 genes identified through univariate Cox analysis that were associated with survival in the TCGA-ESCA dataset, we found that 156 genes were also included among the potential SDGs. To investigate the relationship between SDGs and prognosis in esophageal cancer patients, we applied the LASSO algorithm to the training set. The regularization parameter (*λ*) was determined through 10-fold cross-validation: dividing the training set into 10 subsets, training the model on 9 subsets, validating on 1 subset, testing different *λ* values, and selecting the *λ* that minimized the mean squared error on the validation set, leading to the identification of 7 key genes (Figs. 3A, B). Through multivariate Cox regression analysis, we ultimately identified four genes and developed a risk scoring model based on these genes. Among the four genes, the hazard ratio (HR) for CPSF6 is 4.3 (95% CI: 1.32–13.9), with a P value of 0.016; the HR for IGBP1 is 3.4 (95% CI: 1.52–7.6), with a P value of 0.003; the HR for MTG2 is 2.9 (95% CI: 0.89–9.7), with a P value of 0.078; and the HR for TCP1 is 3.8 (95% CI: 1.39–10.5), with a P value of 0.009 (Fig. 3C). Using this risk model, patients in the training group were categorized into high-risk and low-risk groups. Figure 3D presents a circular schematic illustrating the chromosomal locations of four genes associated with esophageal cancer prognosis: CPSF6, IGBP1, MTG2, and TCP1.CPSF6, located on chromosome 12, is involved in cellular physiological regulation. IGBP1 resides on the X chromosome and may have functions influenced by specific genetic patterns. MTG2, positioned on chromosome 20, is linked to cellular growth and differentiation. TCP1, found on chromosome 6, plays a role in immune response. These genes affect esophageal cancer development and prognosis via the functional roles associated with their respective chromosomal locations. Additionally, Fig. 3E illustrates the risk score rankings of patients in the training set, while Fig. 3F depicts the survival status of each patient, revealing a significantly higher mortality rate in the high-risk group. These findings underscore the potential of the identified genes as prognostic biomarkers in esophageal cancer. Kaplan-Meier survival analysis revealed that the overall survival rate (OS) for the high-risk group was significantly lower compared to the low-risk group (Fig. 3G). Figure 3H presents a heatmap illustrating the expression patterns of four genes—CPSF6, IGBP1, MTG2, and TCP1—in high- and low-risk esophageal cancer groups. The heatmap reveals that in high-risk samples, these genes predominantly display red or reddish hues, indicating elevated expression levels. Conversely, in low-risk samples, they mainly show blue or bluish hues, reflecting lower expression levels. To verify the model’s reliability, we performed validation on multiple datasets. In the test group (*n* = 78, Fig. 4A), the TCGA-ESCA cohort (*n* = 157, Fig. 4B), GSE 254,942 (*n* = 106, Fig. 4C), and GSE 53,624 (*n* = 238, Fig. 4D), from scatter plots and risk score change curves to survival curves, all results indicated that the overall survival rate of the high-risk group was significantly lower than that of the low-risk group (all *P* < 0.05). Notably, the hazard ratio (HR) values for the four genes CPSF6, IGBP1, MTG2, and TCP1 in the forest plot were all greater than 1, indicating significantly elevated expression levels in the high-risk group (Figs. 3H and 4E). To improve the clarity of our findings, we provide a brief overview of the biological functions of the four identified genes. CPSF6 (cleavage and polyadenylation specificity factor 6) is involved in the 3’-end processing of pre-mRNA, which regulates the stability of gene expression. Dysregulation of CPSF6 has been linked to cancer cell proliferation and metastasis. IGBP1 (insulin-like growth factor-binding protein 1) modulates the activity of insulin-like growth factors, influencing cellular metabolism, proliferation, and survival. MTG2 (myeloid translocation gene 2) functions as a transcriptional regulator and is involved in chromatin remodeling and gene expression regulation, playing established roles in hematopoietic development and tumor suppression. TCP1 (T-complex protein 1), a member of the chaperone family, facilitates proper protein folding and helps maintain proteostasis. Abnormal expression of TCP1 has been associated with tumor cell proliferation and drug resistance. Understanding these molecular functions offers mechanistic insights into the potential contributions of these genes to esophageal cancer prognosis.

### Independent variable analysis for disease prognosis

**Fig. 5:**
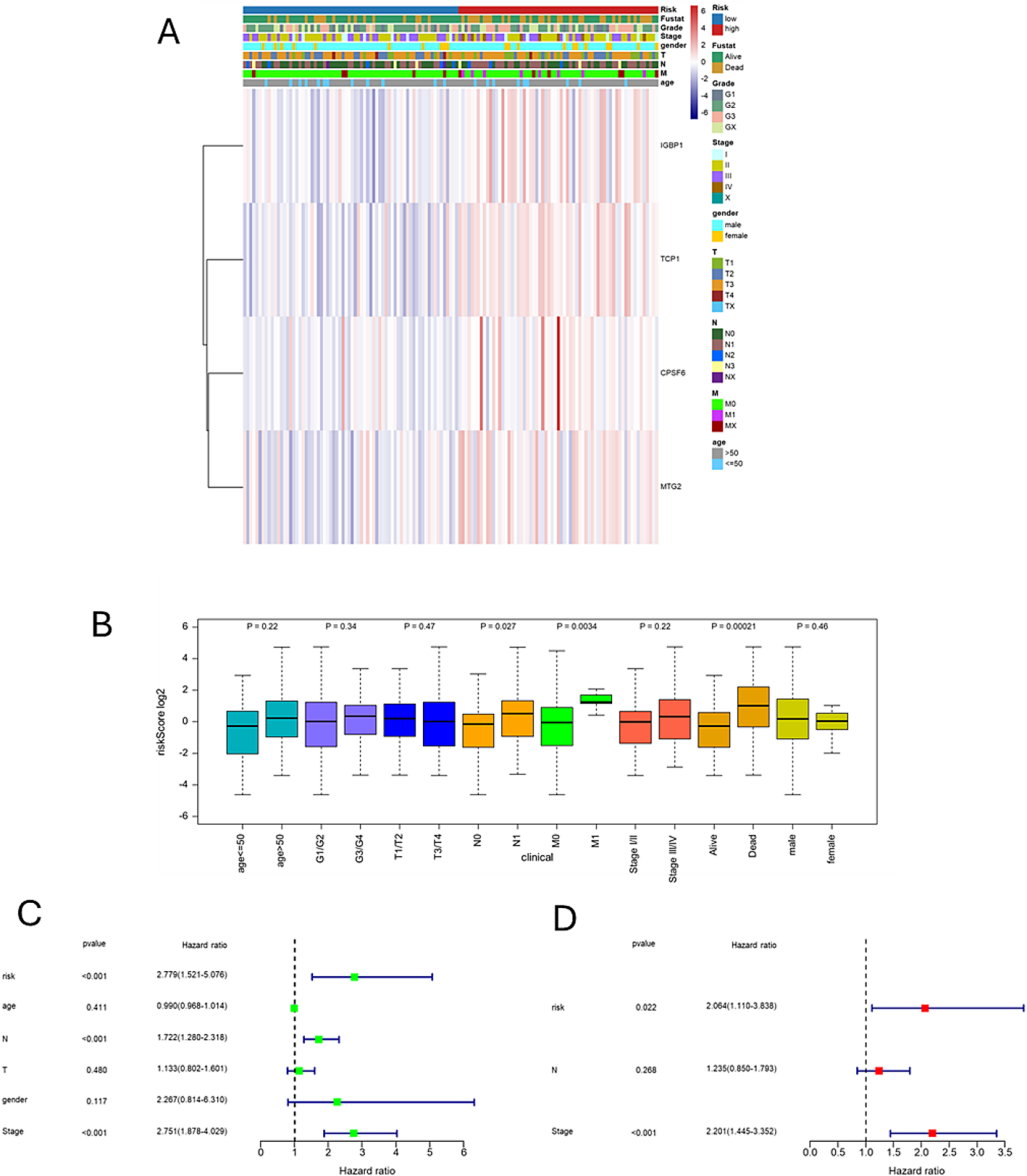
Clinical data from TCGA-ESCA along with independent prognostic factors (**A**) In the TCGA-ESCA dataset, this figure illustrates the expression levels of four genes (IGBP1, TCP1, CPSF6, and MTG2) in conjunction with various clinical characteristics of the samples, including risk class, grade, stage, sex, T stage, N stage, M stage, and age. Different colors denote distinct classifications, with the color intensity in the heatmap reflecting the gene expression levels (**B**) Some patients grouped according to clinical information are compared with riskscore (**C**) Univariate Cox analysis of survival for Risk, Stage, Age and Primary (**D**) Multivariate Cox analysis of survival for risk and stage

We presented the distribution of high-risk and low-risk patients across various subgroups of TCGA-ESCA (Fig. 5A). Figure 5B shows that the risk score features are significantly correlated with patient status (*P* = 0.00021), N stage (*P* = 0.027), and M stage (*P* = 0.0034). Kaplan-Meier analysis indicates that in subgroups such as age ≤ 60 (*P* = 0.0042), > 60 (*P* = 0.037), male (*P* < 0.0001), T3 + T4 (*P* = 0.00035), stage III + IV (*P* = 0.0092), and G2 + G3 (*P* = 0.0032), high-risk patients (red curve) have significantly worse survival. In subgroups such as female (*P* = 0.37), T1 + T2 (*P* = 0.14), N- (*P* = 0.52), N+ (*P* = 0.056), M0 (*P* = 0.1), M1 (*P* = 0.13), stage I + II (*P* = 0.29), and G1 (*P* = 0.99), no significant survival difference was observed between risk groups, or differences were marginally close to significance (Supplementary Fig S1). This may be due to limited sample size; future studies should consider larger cohorts for validation. Following this, we conducted univariate and multivariate Cox analyses within the TCGA cohort to evaluate whether the risk scoring model serves as an independent prognostic factor for overall survival. Figure 5C and D present the results of univariate and multivariate Cox analyses, respectively. In Fig. 5C, the univariate Cox analysis indicates that the risk factor (risk score) has a P-value of less than 0.001 and a hazard ratio (HR) of 2.779 (95% confidence interval: 1.521–5.076), demonstrating a significant association with mortality risk. The tumor stage factor also shows a significant association, with a P-value of less than 0.001 and an HR of 2.751 (95% CI: 1.878–4.029).In contrast, factors such as age and others do not exhibit a statistically significant association with overall survival. Figure 5D presents the results of the multivariate Cox analysis, indicating that the risk factor has a P-value of 0.022 and a hazard ratio (HR) of 2.064 (95% CI: 1.110–3.838), confirming it as a significant independent prognostic factor for overall survival. The tumor stage factor has a P-value of less than 0.001 and an HR of 2.201 (95% CI: 1.445–3.352), further demonstrating a significant independent effect. In contrast, the lymph node metastasis status (N factor) has a P-value of 0.268, indicating no significant independent effect. Collectively, these results confirm that the risk score model can act as an independent prognostic factor for overall survival in patients with esophageal cancer, aiding in the stratification of patient survival.

### Nomogram Building and assessment

**Fig. 6:**
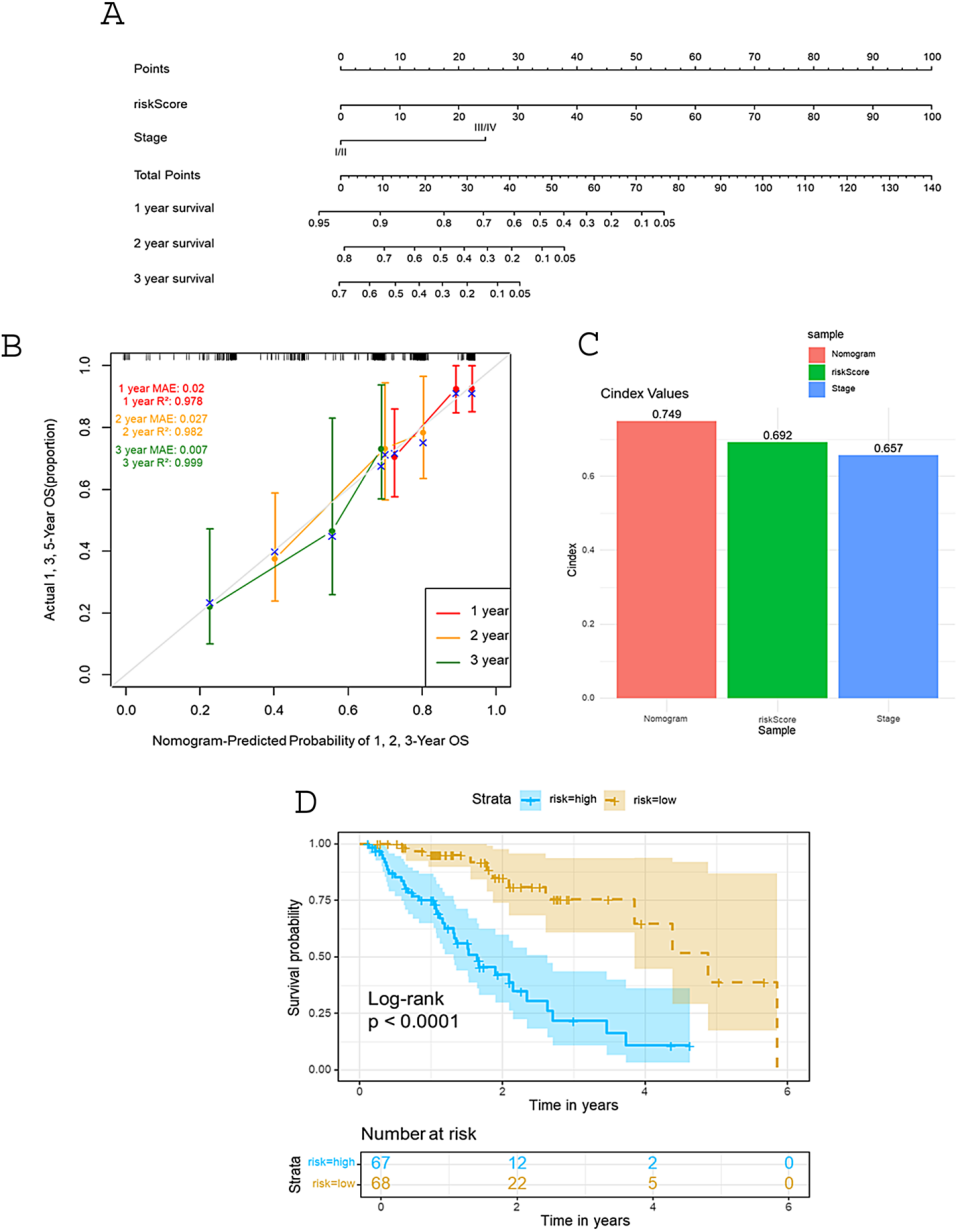
Nomogram model construction and validation (**A**) Nomogram was used to assess the probability of survival of esophageal carcinoma patients based on risk score, stage (**B**) Calibration curves of the nomogram predicting the overall survival (OS) at 1-, 2-, and 3-year intervals in the TCGA ESCA dataset (**C**) The C-Index value of the riskScore and Stage was utilized to analyze the concordance index of the nomogram, evaluating the predictive model’s agreement between its predictions and actual outcomes **(D)** The results of survival analysis based on risk stratification from the nomogram

To quantitatively predict the survival probability of esophageal cancer patients using four genes as prognostic features, we constructed a prognostic nomogram based on Cox proportional hazards regression analysis to estimate 1-year, 2-year, and 3-year overall survival (OS) (Fig. 6A). The nomogram is calculated based on the Cox proportional hazards regression model, with the weighted score for each predictor variable determined by the regression coefficients. These scores are summed to yield a comprehensive risk score, where higher scores indicate an increased risk of mortality. Finally, using the predetermined scales of the nomogram, the scores are converted into predicted survival probabilities for 1 year, 2 years, and 3 years, enabling the transformation of multivariable data into personalized survival risk assessments. The calibration curve (Fig. 6B) visually demonstrates the high consistency between the nomogram’s predicted survival probabilities for esophageal cancer patients at 1 year, 2 years, and 3 years and the actual observed outcomes. Quantitative analysis further confirms its excellent performance: the mean absolute error (MAE) for the 1-year prediction is 0.020 with an R² value of 0.978; the MAE for the 2-year prediction is 0.027 with an R² value of 0.982; and the MAE for the 3-year prediction is as low as 0.007 with an R² value reaching 0.999. The MAE reflects the average deviation between predicted and actual values, while the R² quantifies the goodness of fit of the model to the data, both indicating the predictive accuracy of the nomogram at different time points. Additionally, the concordance index results (Fig. 6C) further validate the high efficacy of this nomogram in predicting survival in esophageal cancer. Figure 6D illustrates the survival analysis based on risk stratification derived from the nomogram. Based on the risk scores from the nomogram, patients were categorized into high-risk and low-risk groups. The survival curves of the two groups are clearly separated, with the high-risk group showing a more rapid decline in survival probability over time. The log-rank test reveals a p-value of less than 0.0001, indicating a highly significant difference between the groups. Overall, these findings support the utility of the nomogram as a practical tool for clinical decision-making in esophageal cancer management.

### WGCNA and GSEA

**Fig. 7:**
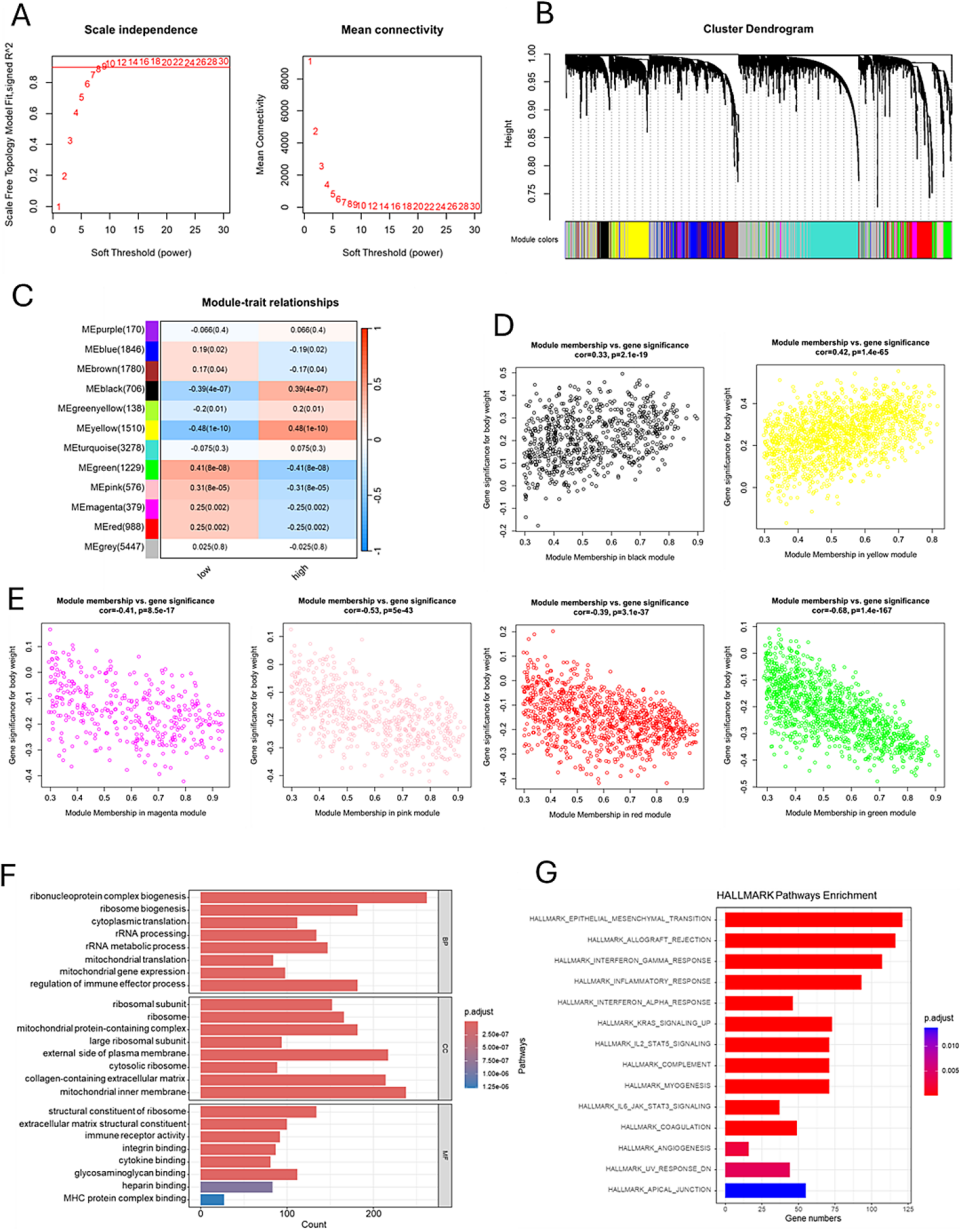
Construction of Weighted Gene Co-Expression Network Analysis (WGCNA) network. (**A**) The scale-free fit index for various soft-thresholding powers. The mean connectivity for various soft-thresholding powers. (**B**) Shows the original and combined modules under the clustering tree (**C**) Heatmap depicting the traits relationship between low- and high-risk groups. (D) MM vs. GS scatter plot of high. E. MM vs. GS scatter plot of low (**F**) KEGG hallmark enrichment analysis. (**G**) GO enrichment analysis based on biological processes (BP), cellular compartments (CC), and molecular functions (MF)

**Fig. 8:**
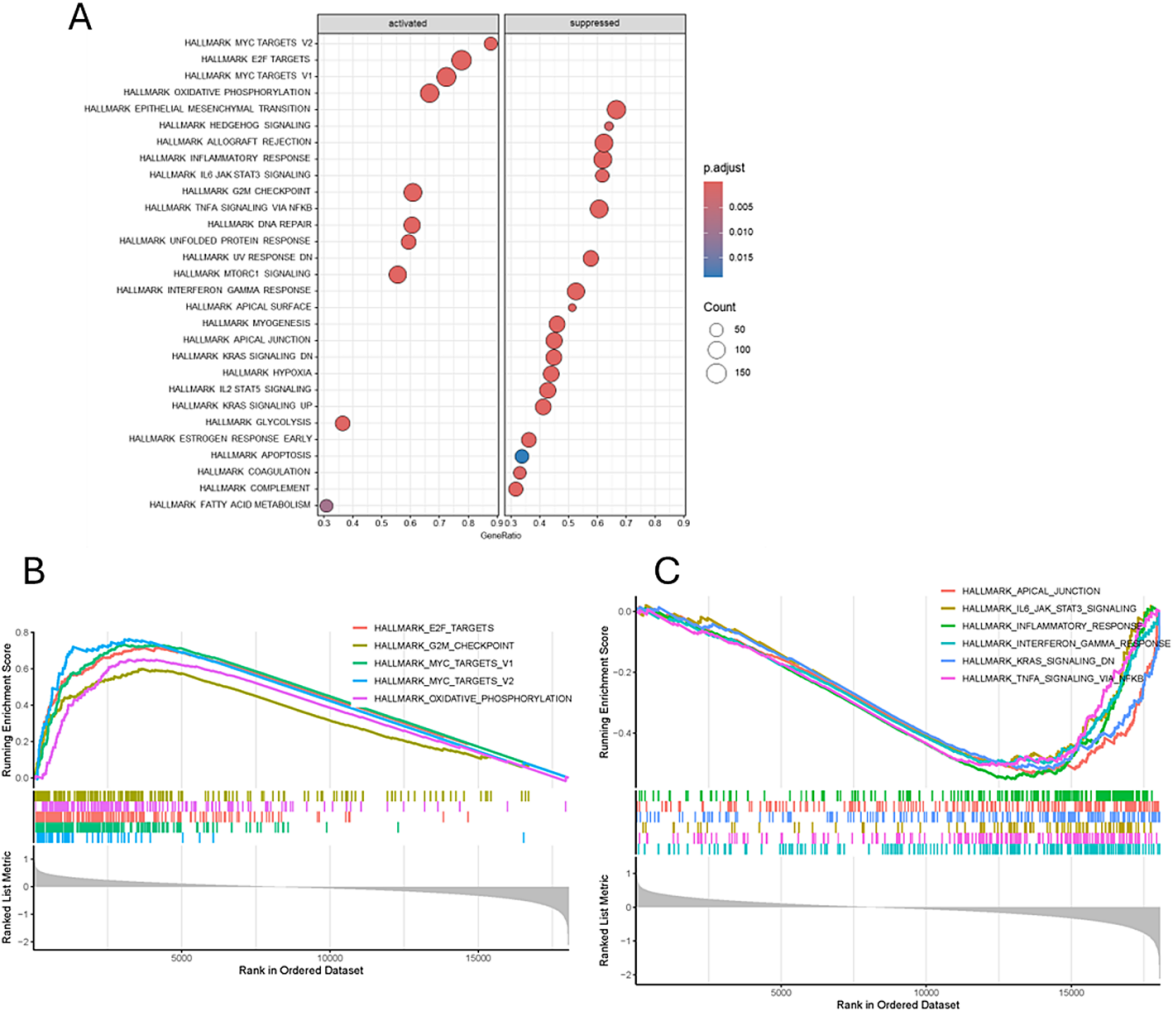
Gene Set Enrichment Analysis (GSEA) of high - and low-risk groups in the TCGA-ESCA dataset. Panel A shows the enrichment results with adjusted p-values indicated; Panels B and C display the running enrichment scores across ranked gene lists for different gene sets

To explore the mechanisms of high-risk and low-risk prognoses in esophageal cancer, we utilized weighted gene co-expression network analysis (WGCNA) to identify co-expressed genes. We divided patients into high-risk and low-risk groups based on the risk scores of TCGA-ESCA patients and performed clustering of the included samples using the average linkage hierarchical clustering method. In constructing the scale-free network, a soft threshold parameter of b = 8 was selected. As shown in Fig. 7A, this value yields a high R² of 0.9 in the “Scale independence” plot, indicating strong scale-free characteristics. Additionally, in the “Mean connectivity” plot, the average connectivity is maintained within an appropriate range, balancing gene connections and network density to ensure the quality of subsequent analyses. Through average linkage hierarchical clustering, a total of 12 modules were determined (Fig. 7B). It was observed that different modules were associated with the high-risk population (Fig. 7C), particularly the black and yellow modules which were positively correlated with high risk (Fig. 7D), while the magenta, pink, red, and green modules were negatively correlated with high risk (Fig. 7E). The genes from these modules were combined for Gene Ontology (GO) analysis. The results of the GO analysis revealed (Fig. 7F) that these genes were involved in the regulation of immune effector processes, immune receptor activity, and cytokine functions, as well as biogenesis of ribonucleoprotein complexes, ribosome biogenesis, cytoplasmic translation, and rRNA processing. We performed HALLMARK enrichment analysis on the genes from the magenta, pink, red, and green modules, identifying key pathways related to immune responses in the HALLMARK analysis (Fig. 7G), including allograft rejection, interferon-gamma response, interferon-alpha response, and inflammatory response. In addition, gene set enrichment analysis (GSEA) was performed on gene sets in the high-risk and low-risk groups from the Cancer Genome Atlas (TCGA)-esophageal squamous cell carcinoma (ESCA) dataset, and multiple gene sets related to immune responses were identified. Figure 8A shows the distribution of gene set enrichment scores for various gene sets. Among these gene sets, we found that immune-related pathways such as interferon - gamma response, il6 jak stat3 signaling pathway, inflammatory response, coagulation, complement, and allograft rejection are suppressed. In Fig. 8B and C, it is observed that these gene sets exhibit inhibitory effects in the high-risk group compared to the low-risk group.

### Determine the link between risk score and immune infiltration patterns

**Fig. 9:**
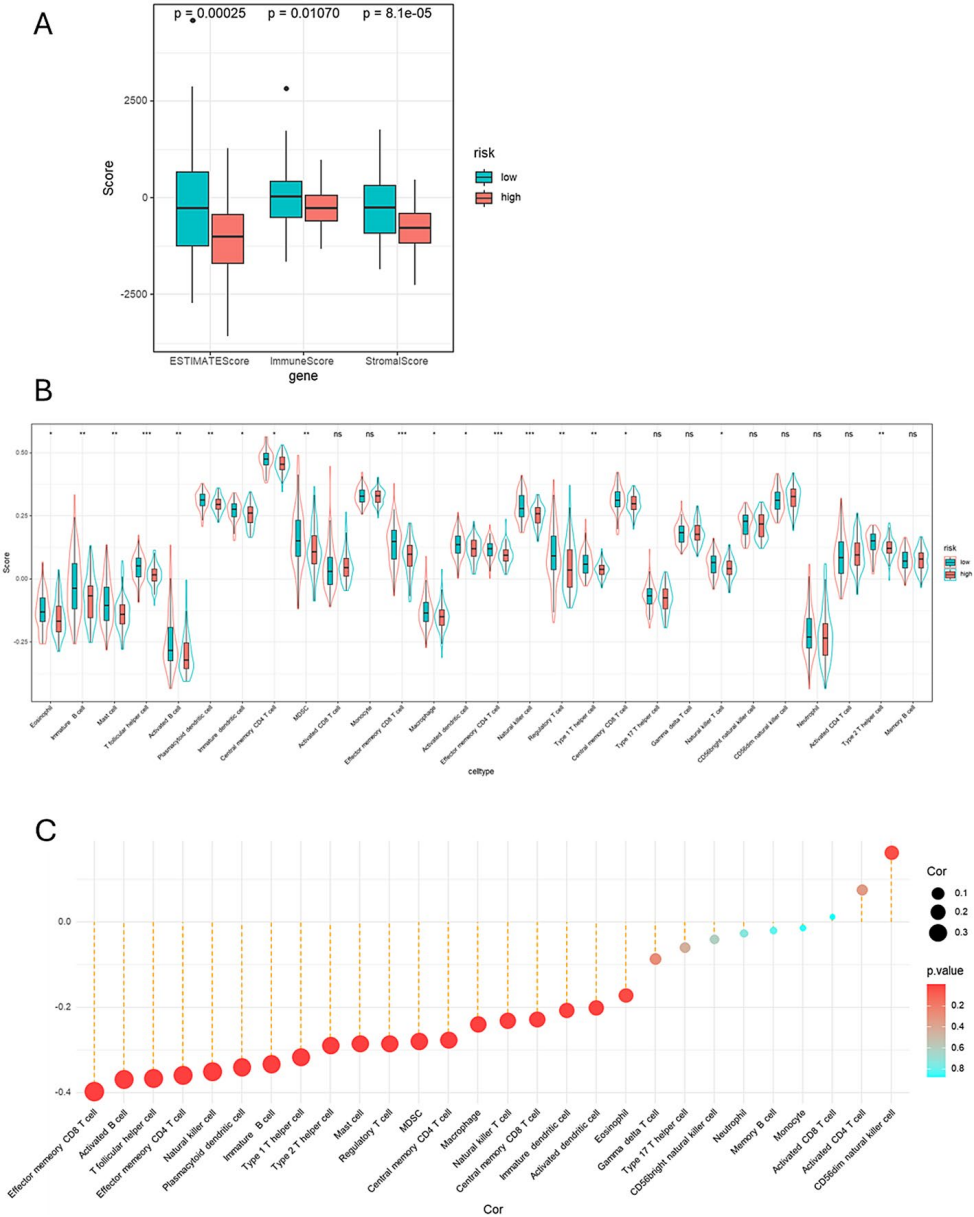
Correlation between risk score and immune infiltration in ESCA (**A**) Differences in ESTIMATE score, immune score, and stromal score between the high-risk and low-risk groups. (**B**) Analyzing immune cell type variations among high-risk vs. low-risk cohorts for 28 distinct types. (**C**) Investigating the link between risk scores and 28 immune cell types

We further compared immune scores, stromal scores, and ESTIMATE scores to evaluate the differences in immune status between the high-risk and low-risk groups. As depicted in Fig. 9A, there is a significant difference in ESTIMATE scores between the high-risk and low-risk groups, with a p-value of 0.00025; for immune scores, the p-value for the difference between the two groups is 0.01070; and in the stromal score comparison, the p-value for the difference between the two groups is 8.1e-05. These results suggest that the immune scores, stromal scores, and ESTIMATE scores are significantly lower in the high-risk population. This finding suggests that a lower immune status may be associated with a poorer prognosis in esophageal cancer patients, highlighting the potential role of the immune microenvironment in disease progression.

To gain a deeper understanding of the relationship between risk scores and tumor-infiltrating immune cells, we conducted single-sample gene-set enrichment analysis (ssGSEA).As illustrated in Fig. 9B, our analysis showed that the infiltration levels of 19 types of immune cells were significantly lower in the high-risk group. The affected immune cells include Eosinophil, Immature B cell, Mast cell, T follicular helper cell, Activated B cell, Plasmacytoid dendritic cell, Immature dendritic cell, Central memory CD4 T cell, MDSC, Effector memory CD 8 T cell, Macrophage, Activated dendritic cell, Effector memory CD4 T cell, Natural killer cell, Regulatory T cell, Type 1 T helper cell, Central memory CD8 T cell, Natural killer T cell, Type 2 T helper cell. This finding underscores the significant differences in immune cell infiltration within the tumor microenvironment between the high-risk and low-risk groups. The results indicate that the decreased presence of immune cells in the tumor microenvironment may contribute to the greater invasiveness observed in high-risk tumors, potentially leading to adverse effects on patient prognosis. Correlation analysis revealed that the risk score was negatively correlated with 25 of the 28 immune cell types, while exhibiting a positive correlation with three immune cell types: Activated CD8 T cells, Activated CD4 T cells, and CD56dim natural killer cells (Fig. 9C). Notably, these findings suggest a potential association between tumor immune infiltration and the poor prognosis observed in the high-risk population. This indicates that the composition of the immune cell landscape may play a crucial role in determining patient outcomes in esophageal cancer.

## Discussion

Esophageal cancer is one of the least understood cancers in the world [10]. Most patients are diagnosed with esophageal cancer at intermediate or advanced stages, primarily because they exhibit no distinct early clinical symptoms. As a result, distant metastasis is frequently present at the time of diagnosis [15]. Although new treatment modalities, including nanomedicine [16] and immunotherapy [17], have emerged, the 5-year survival rate for patients with esophageal cancer remains low, with an overall survival (OS) rate of less than 20%.However, improved outcomes can be observed in patients with early-stage disease [18–20]. Achieving early diagnosis and effective treatment remains a challenge. The identification of new biomarkers could facilitate prognosis assessment and enable the screening of high-risk patients for personalized treatment.

This study innovatively constructs a four-gene prognostic signature comprising CPSF6, IGBP1, MTG2, and TCP1 through CRISPR-Cas9 functional screening and multi-omics integrative analysis. Validated across multiple cohorts, including TCGA and GEO, this model demonstrates excellent risk stratification capability (HR = 2.87, *P* < 0.001) and achieves a C-index of 0.73, indicating an enhancement over traditional single-gene biomarkers. Mechanistic studies reveal that pro-cancer immune pathways, including IL6-JAK-STAT3 and IFN-γ, are significantly suppressed in high-risk patients (NES < -1.5, *P* < 0.01), while the infiltration levels of 19 key immune cells, such as CD8 + T cells and dendritic cells, are diminished in the tumor microenvironment, creating a vicious cycle of immune evasion and immune suppression. In this context, these patients are more likely to experience optimal clinical benefits from a comprehensive regimen that combines immune checkpoint inhibitors with targeted therapy. By blocking immune evasion pathways and restoring immune cell function, this approach is anticipated to overcome tumor immune suppression and introduce new therapeutic strategies aimed at enhancing patient prognosis. This innovative achievement holds dual significance. First, the four-gene prognostic feature model serves as an accurate assessment tool for esophageal cancer patients, effectively distinguishing between high-risk and low-risk populations. Second, the research elucidates the molecular mechanisms underlying immune suppression, establishing a robust theoretical foundation for targeted interventions against tumor immune evasion and the optimization of immunotherapy strategies. This is anticipated to foster further advancements in precision treatment for esophageal cancer.

Numerous studies on esophageal cancer prognosis have investigated molecular biomarkers and associated signaling pathways. For instance, Zhang et al. identified *EFNA1* as significantly linked to esophageal cancer prognosis through large-scale genomic sequencing and bioinformatics analysis [21]. Li et al. proposed *BIN3* as a potential prognostic biomarker in ESCA [22], while Lin et al. highlighted the important role of *RSPO1* in regulating tumor immunity and suggested *RSPO1* as a promising target for immunotherapy in ESCA [23], Deng et al. developed nomograms and risk classification models to predict the prognosis of patients with esophageal squamous cell carcinoma [24]. Research conducted by Tian et al. demonstrates that the identified 5-mRNA prognostic features can reliably predict the prognosis of patients with EAC and support individualized treatment decisions [25]. Nonetheless, these studies have some shortcomings. First, the performance of individual genes in prognostic evaluation is somewhat limited. Second, some multi-gene prognostic models lack external validation, which may result in lower accuracy and reliability compared to our model.

The results of CRISPR/Cas9 genome-wide loss-of-function screening are now publicly available on DepMap portal, encompassing 1,865 cancer cell lines. The DepMap database integrates gene knockouts based on CRISPR-Cas9 and RNAi across various cell lines [26]. Additionally, a computational method named CERES has been developed to estimate gene dependency levels from CRISPR-Cas9 essentiality screenings [27]. CERES reflects the importance of genes for cell survival or proliferation, which is influenced by genotype, gene expression, and cell lineage [28].

The DepMap database has been utilized for the characterization of malignant tumors, pan-cancer datasets, predetermined biological processes, and the development of prognostic models [29]. Its establishment is crucial for advancing research on cancer therapeutic targets [30].

This study integrated results from CRISPR-Cas9 screening for esophageal cancer obtained from DepMap with the TCGA dataset, identifying 156 potential survival-dependent genes. Subsequently, we employed LASSO Cox regression analysis and multivariable Cox regression analysis to select four survival-dependent genes from the training set, thereby establishing a robust 4-gene prognostic signature comprising *CPSF6*, *IGBP1*, *MTG2*, and *TCP1*. Kaplan-Meier analysis revealed significant differences between the two risk groups, with the high-risk group exhibiting poorer outcomes than the low-risk group. We validated these findings using both the test cohort and the entire cohort, as well as the GSE 254,942 and GSE 53,624 validation sets. Additionally, we developed a nomogram model by integrating the prognostic signature with other relevant clinical factors. Our risk score exhibited strong consistency and superior predictive capability compared to other clinical features, as validated by calibration plots.

Among the four genes mentioned above, Immunoglobulin Binding Protein 1 (*IGBP1*) is an important signaling regulator that mediates various functions. Increased expression of *IGBP1* has been reported to be associated with poor prognosis in ESCC, and *IGBP1* may act as a tumor promoter in ESCC [31]. Additionally, the *TCP1* subunit 6 A (*CCT6A*), which contains chaperone proteins, promotes various malignant cancer behaviors, and *CCT6A* enhances malignant activities in ESCC by activating the *TGF-β/Smad/c-Myc* pathway [32]. Although there is a lack of studies examining the effects of the remaining two genes on ESCA, they have been shown to be associated with the development of other cancer types. Cleavage and Polyadenylation Specificity Factor Subunit 6 (*CPSF6*, also known as *CFIm68*) plays a crucial role in selective polyadenylation and is a 68 kDa component of the mammalian Cleavage Factor I (*CFIm*) complex, which regulates mRNA selective polyadenylation (APA) and determines the length of the 3’ untranslated region (UTR), an important mechanism of gene expression control [33]. Reports indicate that *CPSF6* enhances tumorigenic and metastatic activities through different molecular mechanisms and exerts oncogenic effects15. *CPSF6*-mediated shortening of the *XBP1* 3’ UTR alleviates cisplatin-induced ER stress and enhances chemoresistance in lung adenocarcinoma [34]. The effects of non-coding mutations on *MTG2* transcription have been summarized in various cancer cell lines, and increased expression of *DAAM1* leads to aggressive cell migration [35].

To explore the potential molecular mechanisms underlying the significant prognostic differences between high- and low-risk groups in the survival-dependent gene prognostic model, we first analyzed the differences between the two groups using the WGCNA method. Patients were categorized into high-risk and low-risk groups based on their risk scores from TCGA-ESCA, and the included samples were clustered using the average linkage hierarchical clustering method. A soft threshold parameter of b = 8 was chosen to construct a scale-free network, and 12 modules were identified through average linkage hierarchical clustering. The results showed that different modules were associated with the high-risk population, particularly with the black and yellow modules positively correlated with high risk, while the magenta, pink, red, and green modules were negatively correlated with high risk. The genes within these modules were combined for Gene Ontology (GO) analysis. The results of the GO analysis indicate that these genes are associated with the regulation of immune effector processes, immune receptor activity, and cytokine activity, as well as biogenesis of ribonucleoprotein complexes, ribosome biogenesis, cytoplasmic translation, and rRNA processing. We performed HALLMARK enrichment analysis on the genes from the magenta, pink, red, and green modules. The HALLMARK analysis identified key pathways associated with immune responses, including allogeneic graft rejection, interferon-gamma response, interferon-alpha response, and inflammatory response. These immune-related pathways showed inhibitory effects in the high-risk group, suggesting the potential presence of immune suppression in this group. Furthermore, in the gene set enrichment analysis (GSEA) of the TCGA-ESCA dataset, several immune response-related gene sets were identified between the high-risk and low-risk groups. Notably, immune-related pathways such as interferon gamma response, il6 jak stat3 signaling, inflammatory response, coagulation, complement, and allograft rejection showed inhibitory effects. Our immune infiltration analysis revealed that in ESCA patients, the infiltration levels of 19 immune cell types were significantly reduced in the high-risk group. Correlation analysis indicated that the risk score was negatively correlated with 25 out of 28 immune cell types, while it showed positive correlations with 3 immune cell types (Activated CD8 T cell, Activated CD4 T cell, and CD56dim natural killer cell). These results suggest a potential association between tumor immune infiltration and the adverse prognosis observed in the high-risk population. Therefore, the significant prognostic differences are likely due to the differences in immune infiltration between high- and low-risk groups, with immune suppression present in the high-risk group.

Surgical resection, radiotherapy, and chemotherapy are the primary treatment modalities for esophageal cancer. However, the efficacy of these conventional treatments is often limited, and they can cause severe adverse reactions, leading to unsatisfactory outcomes [36]. Immunotherapy represents an effective cancer treatment that can provide an alternative to conventional therapies. Several studies have shown that, in advanced and metastatic esophageal cancer, monotherapy with immunotherapy exhibits superior anti-tumor effects compared to chemotherapy. Furthermore, it has been found that the combination of immunotherapy with chemotherapy is more effective than immunotherapy alone [37].

In various clinical studies centered on immunotherapy, immune checkpoint inhibitors (ICI) have been acknowledged for their potential to enhance survival rates in patients with ESCC [38].For patients with unresectable advanced or locally advanced esophageal cancer and a PD-L1 combined positive score (CPS) ≥ 10, pembrolizumab combined with chemotherapy (5-fluorouracil and cisplatin) shows a significant advantage in overall survival compared to placebo combined with chemotherapy (5-fluorouracil and cisplatin) [39].For patients with locally advanced esophageal adenocarcinoma, studies have shown that the addition of the anti-PD-L1 antibody durvalumab to induction FOLFOX and neoadjuvant chemotherapy is safe. This treatment regimen not only improves the pathological response rate but also demonstrates encouraging survival data [40]. In advanced esophageal squamous cell carcinoma patients, first-line treatment with nivolumab in combination with chemotherapy and first-line treatment with nivolumab combined with ipilimumab both resulted in significantly longer overall survival compared to chemotherapy alone, and no new safety signals were observed [41].

This study showcases significant methodological innovation. By uniquely integrating CRISPR-Cas9 functional screening technology with multi-omics analysis, this distinctive research approach provides broader and more comprehensive perspectives for exploring potential prognostic biomarkers. Utilizing CRISPR-Cas9 functional screening allows for precise dissection of gene functions, while multi-omics integrative analysis incorporates biomolecular information from various dimensions, including genomics and transcriptomics. These two approaches complement one another, significantly increasing the likelihood of identifying key prognostic biomarkers. Furthermore, we validated our findings using multiple large cohorts from TCGA, GEO, and other sources, effectively enhancing the reliability and generalizability of our conclusions. This ensures that the research outcomes are applicable not only to specific datasets but also verifiable across a broader range of clinical samples.

Although this study conducted a comprehensive bioinformatics analysis using TCGA and GEO databases, several limitations must be acknowledged. First and foremost, the data primarily originate from these public databases, with samples predominantly representing specific ethnicities and geographic regions, which may restrict the generalizability of the findings. Furthermore, although we have identified significant activation of pro-cancer immune pathways, such as IL6-JAK-STAT3 and IFN-γ, in high-risk patients, along with a decrease in the infiltration levels of 19 critical immune cells—including CD8 + T cells and dendritic cells—in the tumor microenvironment, we have only begun to elucidate the malignant cycle between immune evasion and immune suppression. The intricate regulatory mechanisms between immune pathways and immune cells are not yet fully understood and require further exploration. In terms of clinical translation, a notable gap remains between current research findings and practical clinical applications, with the absence of prospective clinical study validation serving as a significant barrier. The lack of prospective studies makes it challenging to accurately assess the effectiveness and applicability of the four-gene prognostic signature model in real-world clinical settings. Moreover, the quality and completeness of the database data have impacted the research process. The original dataset contains insufficient treatment record information, with less than 50% of samples annotated with treatment data, hindering our ability to perform a comprehensive treatment history subgroup analysis. Treatment information is crucial for understanding the impact of various treatment regimens on patient prognosis; its absence restricts our ability to further analyze and interpret the research findings, potentially resulting in the oversight of certain associations.

## Electronic supplementary material

Supplementary Material 1

## Data Availability

the data we utilized are derived from well-known public databases, namely TCGA(ESCA), GEO(GSE254942 and GSE53624), and DEPMAP. These databases have robust ethics governance procedures in place.

## References

[1] Bray F, Laversanne M, Sung H, Ferlay J, Siegel RL, Soerjomataram I, et al. Global cancer statistics 2022: GLOBOCAN estimates of incidence and mortality worldwide for 36 cancers in 185 countries. CA Cancer J Clin. 2024;74:229–63. 10.3322/caac.21834. 38572751 10.3322/caac.21834

[2] Zhu H, Ma X, Ye T, Wang H, Wang Z, Liu Q, et al. Esophageal cancer in china: practice and research in the new era. Int J Cancer. 2023;152:1741–51. 10.1002/ijc.34301. 36151861 10.1002/ijc.34301

[3] Smyth EC, Lagergren J, Fitzgerald RC, Lordick F, Shah MA, Lagergren P, et al. Oesophageal cancer. Nat Rev Dis Primer. 2017;3:17048. 10.1038/nrdp.2017.48. 10.1038/nrdp.2017.48 PMC6168059 28748917

[4] Sun D, Liu C, Zhu Y, Yu C, Guo Y, Sun D, et al. Long-Term exposure to fine particulate matter and incidence of esophageal cancer: A prospective study of 0.5 million Chinese adults. Gastroenterology. 2023;165:61–e705. 10.1053/j.gastro.2023.03.233. 37059339 10.1053/j.gastro.2023.03.233 PMC7615725

[5] Deboever N, Jones CM, Yamashita K, Ajani JA, Hofstetter WL. Advances in diagnosis and management of cancer of the esophagus. BMJ. 2024;385:e074962. 10.1136/bmj-2023-074962. 38830686 10.1136/bmj-2023-074962

[6] Mansour NM, Groth SS, Anandasabapathy S. Esophageal adenocarcinoma: screening, surveillance, and management. Annu Rev Med. 2017;68:213–27. 10.1146/annurev-med-050715-104218. 27618753 10.1146/annurev-med-050715-104218

[7] Xie S-H, Lagergren J. The male predominance in esophageal adenocarcinoma. clin gastroenterol hepatol off clin pract. J Am Gastroenterol Assoc. 2016;14:338–e3471. 10.1016/j.cgh.2015.10.005. 10.1016/j.cgh.2015.10.005 26484704

[8] Kurumi H, Isomoto H. Current topics in esophageal squamous cell carcinoma. Cancers. 2020;12:2898. 10.3390/cancers12102898. 33050257 10.3390/cancers12102898 PMC7601260

[9] DaSilva LL, Aguiar PN, de Lima Lopes G. Immunotherapy for advanced esophageal squamous cell Carcinoma-Renewed enthusiasm and a lingering challenge. JAMA Oncol. 2021;7:1613–4. 10.1001/jamaoncol.2021.4410. 34519775 10.1001/jamaoncol.2021.4410

[10] Yang T, Hui R, Nouws J, Sauler M, Zeng T, Wu Q. Untargeted metabolomics analysis of esophageal squamous cell cancer progression. J Transl Med. 2022;20:127. 10.1186/s12967-022-03311-z. 35287685 10.1186/s12967-022-03311-z PMC8919643

[11] Joseph A, Raja S, Kamath S, Jang S, Allende D, McNamara M, et al. Esophageal adenocarcinoma: A dire need for early detection and treatment. Cleve Clin J Med. 2022;89:269–79. 10.3949/ccjm.89a.21053. 35500930 10.3949/ccjm.89a.21053

[12] Rubenstein JH, Shaheen NJ. Epidemiology, diagnosis, and management of esophageal adenocarcinoma. Gastroenterology. 2015;149:302. 10.1053/j.gastro.2015.04.053. 25957861 10.1053/j.gastro.2015.04.053 PMC4516638

[13] Shi B, Ding J, Qi J, Gu Z. Characteristics and prognostic value of potential dependency genes in clear cell renal cell carcinoma based on a large-scale CRISPR-Cas9 and RNAi screening database DepMap. Int J Med Sci. 2021;18:2063–75. 10.7150/ijms.51703. 33850477 10.7150/ijms.51703 PMC8040392

[14] Mijiti M, Maimaiti A, Chen X, Tuersun M, Dilixiati M, Dilixiati Y, et al. CRISPR-cas9 screening identified lethal genes enriched in Hippo kinase pathway and of predictive significance in primary low-grade glioma. Mol Med Camb Mass. 2023;29:64. 10.1186/s10020-023-00652-3. 37183261 10.1186/s10020-023-00652-3 PMC10183247

[15] Huang F-L, Yu S-J. Esophageal cancer: risk factors, genetic association, and treatment. Asian J Surg. 2018;41:210–5. 10.1016/j.asjsur.2016.10.005. 27986415 10.1016/j.asjsur.2016.10.005

[16] Li X, Chen L, Luan S, Zhou J, Xiao X, Yang Y, et al. The development and progress of nanomedicine for esophageal cancer diagnosis and treatment. Semin Cancer Biol. 2022;86:873–85. 10.1016/j.semcancer.2022.01.007. 35074509 10.1016/j.semcancer.2022.01.007

[17] Kumagai S, Koyama S, Shitara K. Precise patient stratification in esophageal cancer: biomarkers for immunochemotherapy. Cancer Cell. 2023;41:1199–201. 10.1016/j.ccell.2023.06.004. 37433280 10.1016/j.ccell.2023.06.004

[18] Petrillo A, Smyth EC. Immunotherapy for squamous esophageal cancer: A review. J Pers Med. 2022;12:862. 10.3390/jpm12060862. 35743646 10.3390/jpm12060862 PMC9225249

[19] Li J, Xu J, Zheng Y, Gao Y, He S, Li H, et al. Esophageal cancer: epidemiology, risk factors and screening. Chin J Cancer Res. 2021;33:535. 10.21147/j.issn.1000-9604.2021.05.01. 34815628 10.21147/j.issn.1000-9604.2021.05.01 PMC8580797

[20] Kelly RJ. Emerging multimodality approaches to treat localized esophageal Cancer. J Natl Compr Cancer Netw JNCCN. 2019;17:1009–14. 10.6004/jnccn.2019.7337. 31390584 10.6004/jnccn.2019.7337

[21] Zhang Y, Zhang J, Pan G, Guan T, Zhang C, Hao A, et al. Effects of EFNA1 on cell phenotype and prognosis of esophageal carcinoma. World J Surg Oncol. 2021;19:242. 10.1186/s12957-021-02362-8. 34399788 10.1186/s12957-021-02362-8 PMC8369630

[22] Li D, Deng W, Huang G, Xiao X. Bridging integrator 3 (BIN3) downregulation predicts a poor prognosis in patients with esophagus carcinoma: A study based on TCGA data. Comb Chem High Throughput Screen. 2023;26:1974–89. 10.2174/1386207326666221205101815. 36475339 10.2174/1386207326666221205101815 PMC10332122

[23] Lin Y, Lou X, Li S, Cai W, Che T. Identification and validation of immune implication of R-Spondin 1 and an R-Spondin 1-Related prognostic signature in esophagus Cancer. Int J Genomics. 2024;2024:7974277. 10.1155/2024/7974277. 38962149 10.1155/2024/7974277 PMC11222003

[24] Deng J, Weng X, Chen W, Zhang J, Ma L, Zhao K. A nomogram and risk classification model predicts prognosis in Chinese esophageal squamous cell carcinoma patients. Transl Cancer Res. 2022;11:3128–40. 10.21037/tcr-22-915. 36237263 10.21037/tcr-22-915 PMC9552058

[25] Lan T, Liu W, Lu Y, Luo H. A five-gene signature for predicting overall survival of esophagus adenocarcinoma. Med (Baltim). 2021;100:e25305. 10.1097/MD.0000000000025305. 10.1097/MD.0000000000025305 PMC8036055 33832101

[26] Tsherniak A, Vazquez F, Montgomery PG, Weir BA, Kryukov G, Cowley GS, et al. Defining a Cancer dependency map. Cell. 2017;170:564. 10.1016/j.cell.2017.06.010. 28753430 10.1016/j.cell.2017.06.010 PMC5667678

[27] Meyers RM, Bryan JG, McFarland JM, Weir BA, Sizemore AE, Xu H, et al. Computational correction of copy number effect improves specificity of CRISPR-Cas9 essentiality screens in cancer cells. Nat Genet. 2017;49:1779–84. 10.1038/ng.3984. 29083409 10.1038/ng.3984 PMC5709193

[28] Shimada K, Bachman JA, Muhlich JL, Mitchison TJ. shinyDepMap, a tool to identify targetable cancer genes and their functional connections from Cancer dependency map data. eLife. 2021;10:e57116. 10.7554/eLife.57116. 33554860 10.7554/eLife.57116 PMC7924953

[29] Sannigrahi MK, Cao AC, Rajagopalan P, Sun L, Brody RM, Raghav L, et al. A novel pipeline for prioritizing cancer type-specific therapeutic vulnerabilities using DepMap identifies PAK2 as a target in head and neck squamous cell carcinomas. Mol Oncol. 2024;18:336–49. 10.1002/1878-0261.13558. 37997254 10.1002/1878-0261.13558 PMC10850805

[30] Yang X, Liu J, Wang S, Al-Ameer WHA, Ji J, Cao J, et al. Genome wide-scale CRISPR-Cas9 knockout screens identify a fitness score for optimized risk stratification in colorectal cancer. J Transl Med. 2024;22:554. 10.1186/s12967-024-05323-3. 38858785 10.1186/s12967-024-05323-3 PMC11163718

[31] Jiang S, Li D, Liang Z, Wang Y, Pei X, Tang J. High expression of IGBP1 correlates with poor prognosis in esophageal squamous cell carcinoma. Int J Biol Markers. 2020;35:33–40. 10.1177/1724600819896374. 31875416 10.1177/1724600819896374

[32] Xia X, Zhao S, Chen W, Xu C, Zhao D. CCT6A promotes esophageal squamous cell carcinoma cell proliferation, invasion and epithelial-mesenchymal transition by activating TGF-β/Smad/c-Myc pathway. Ir J Med Sci. 2023;192:2653–60. 10.1007/s11845-023-03357-y. 37017854 10.1007/s11845-023-03357-y

[33] Bialas K, Diaz-Griffero F. HIV-1-induced translocation of CPSF6 to biomolecular condensates. Trends Microbiol. 2024;32:781–90. 10.1016/j.tim.2024.01.001. 38267295 10.1016/j.tim.2024.01.001 PMC11263504

[34] Zhu C, Xie Y, Li Q, Zhang Z, Chen J, Zhang K, et al. CPSF6-mediated XBP1 3’UTR shortening attenuates cisplatin-induced ER stress and elevates chemo-resistance in lung adenocarcinoma. Drug Resist Updat Rev Comment Antimicrob Anticancer Chemother. 2023;68:100933. 10.1016/j.drup.2023.100933. 10.1016/j.drup.2023.100933 36821972

[35] Zhang W, Bojorquez-Gomez A, Velez DO, Xu G, Sanchez KS, Shen JP, et al. A global transcriptional network connecting noncoding mutations to changes in tumor gene expression. Nat Genet. 2018;50:613. 10.1038/s41588-018-0091-2. 29610481 10.1038/s41588-018-0091-2 PMC5893414

[36] Yang Y-M, Hong P, Xu WW, He Q-Y, Li B. Advances in targeted therapy for esophageal cancer. Signal Transduct Target Ther. 2020;5:229. 10.1038/s41392-020-00323-3. 33028804 10.1038/s41392-020-00323-3 PMC7542465

[37] Wang Y, Yang W, Wang Q, Zhou Y. Mechanisms of esophageal cancer metastasis and treatment progress. Front Immunol. 2023;14:1206504. 10.3389/fimmu.2023.1206504. 37359527 10.3389/fimmu.2023.1206504 PMC10285156

[38] Xiong G, Chen Z, Liu Q, Peng F, Zhang C, Cheng M, et al. CD276 regulates the immune escape of esophageal squamous cell carcinoma through CXCL1-CXCR2 induced NETs. J Immunother Cancer. 2024;12:e008662. 10.1136/jitc-2023-008662. 38724465 10.1136/jitc-2023-008662 PMC11086492

[39] Sun J-M, Shen L, Shah MA, Enzinger P, Adenis A, Doi T, et al. Pembrolizumab plus chemotherapy versus chemotherapy alone for first-line treatment of advanced oesophageal cancer (KEYNOTE-590): a randomised, placebo-controlled, phase 3 study. Lancet Lond Engl. 2021;398:759–71. 10.1016/S0140-6736(21)01234-4. 10.1016/S0140-6736(21)01234-4 34454674

[40] Cowzer D, Wu AJ-C, Sihag S, Walch HS, Park BJ, Jones DR, et al. Durvalumab and PET-Directed chemoradiation in locally advanced esophageal adenocarcinoma: A phase ib/ii study. Ann Surg. 2023;278:e511–8. 10.1097/SLA.0000000000005818. 36762546 10.1097/SLA.0000000000005818 PMC11065504

[41] Doki Y, Ajani JA, Kato K, Xu J, Wyrwicz L, Motoyama S, et al. Nivolumab combination therapy in advanced esophageal Squamous-Cell carcinoma. N Engl J Med. 2022;386:449–62. 10.1056/NEJMoa2111380. 35108470 10.1056/NEJMoa2111380

