# Supplementary material for: Identification and characterization of survival-dependent genes in esophageal cancer via the DepMap database: unraveling their association with immune infiltration
Source: Discov Oncol. 2025 Jun 22;16:1176. doi: 10.1007/s12672-025-02942-0 (PMC12183143; doi:10.1007/s12672-025-02942-0)
Supplement: Supplementary file 1 — Supplementary Material 1 [file 12672_2025_2942_MOESM1_ESM.docx]

Supplementary Figure 1


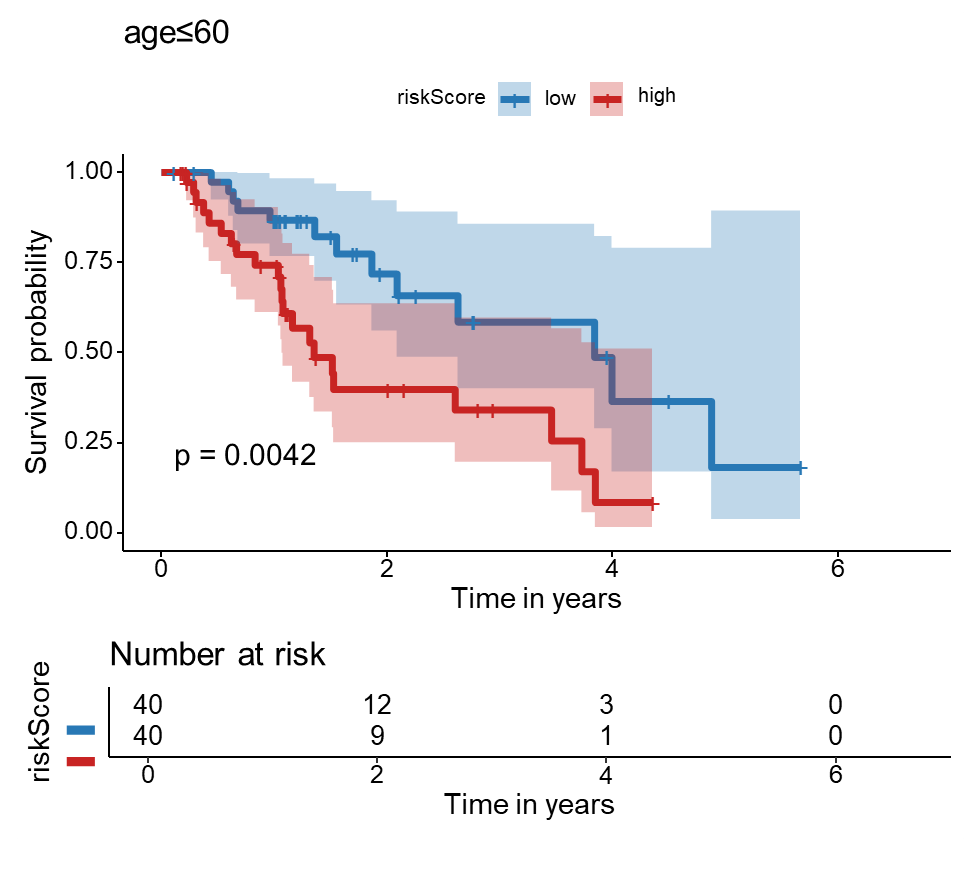

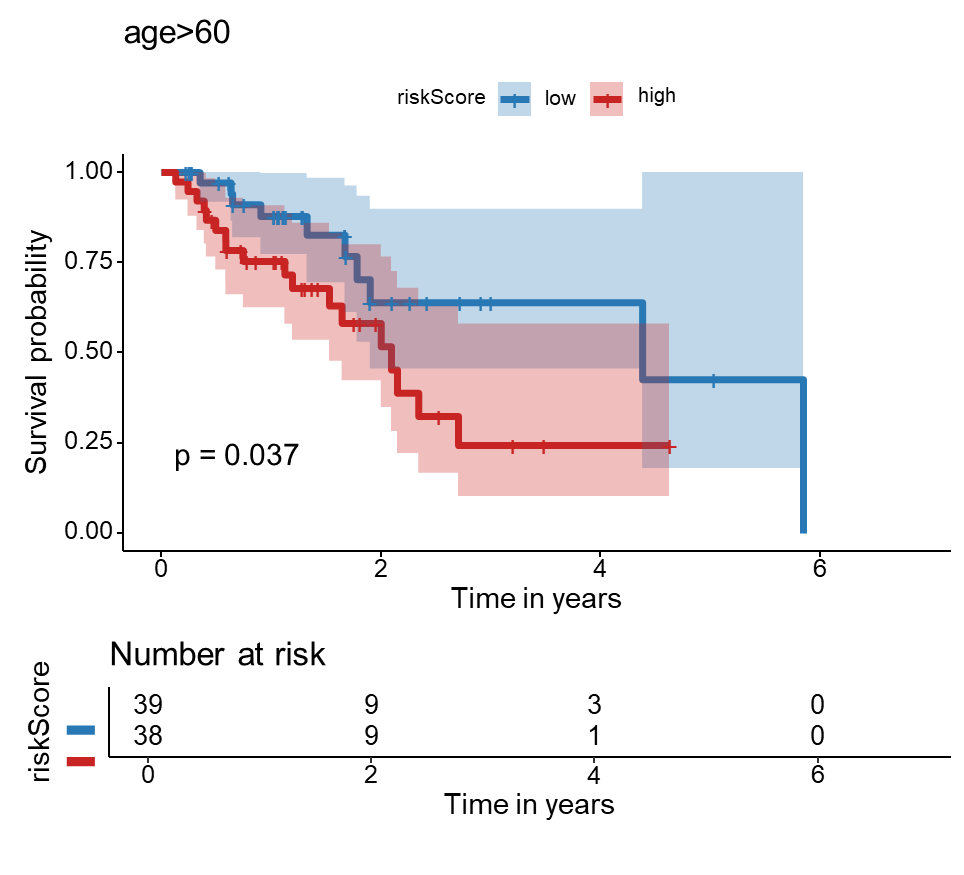

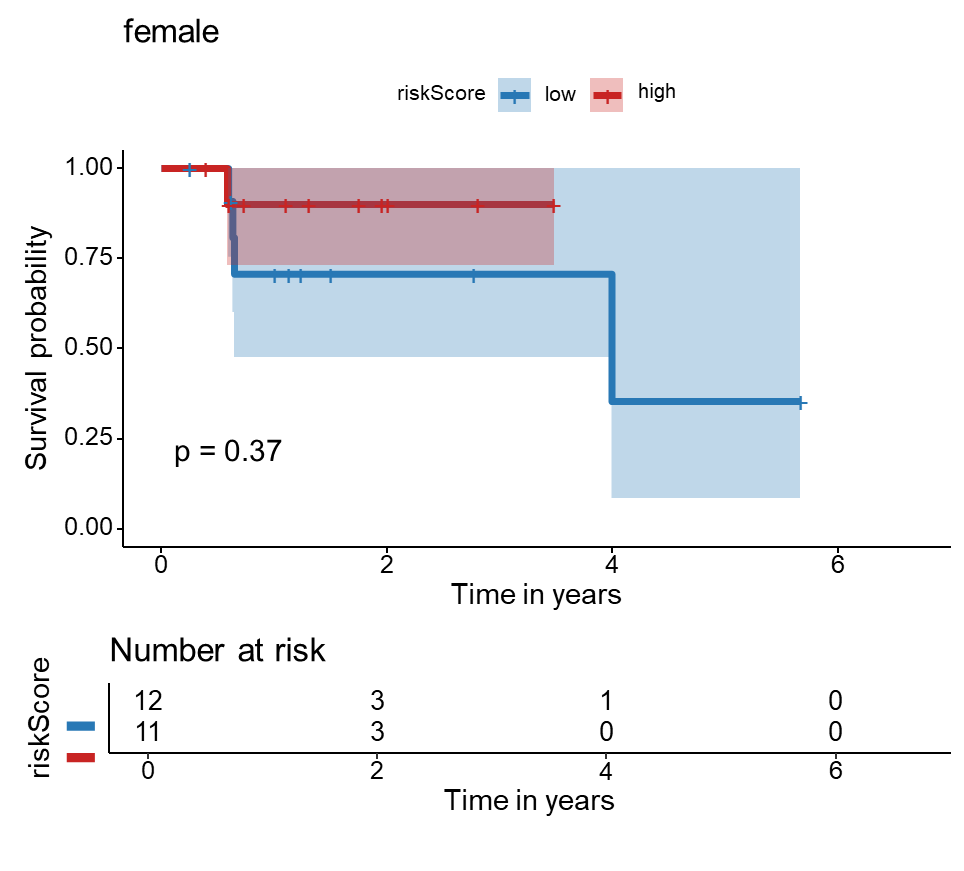

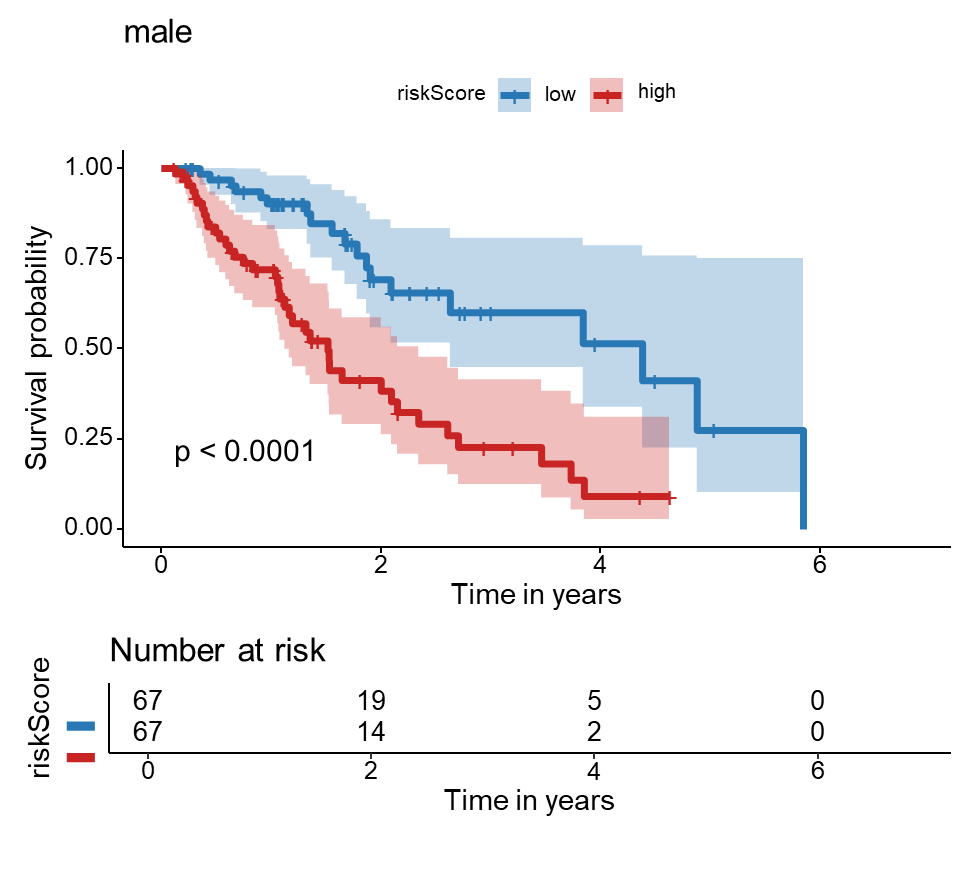

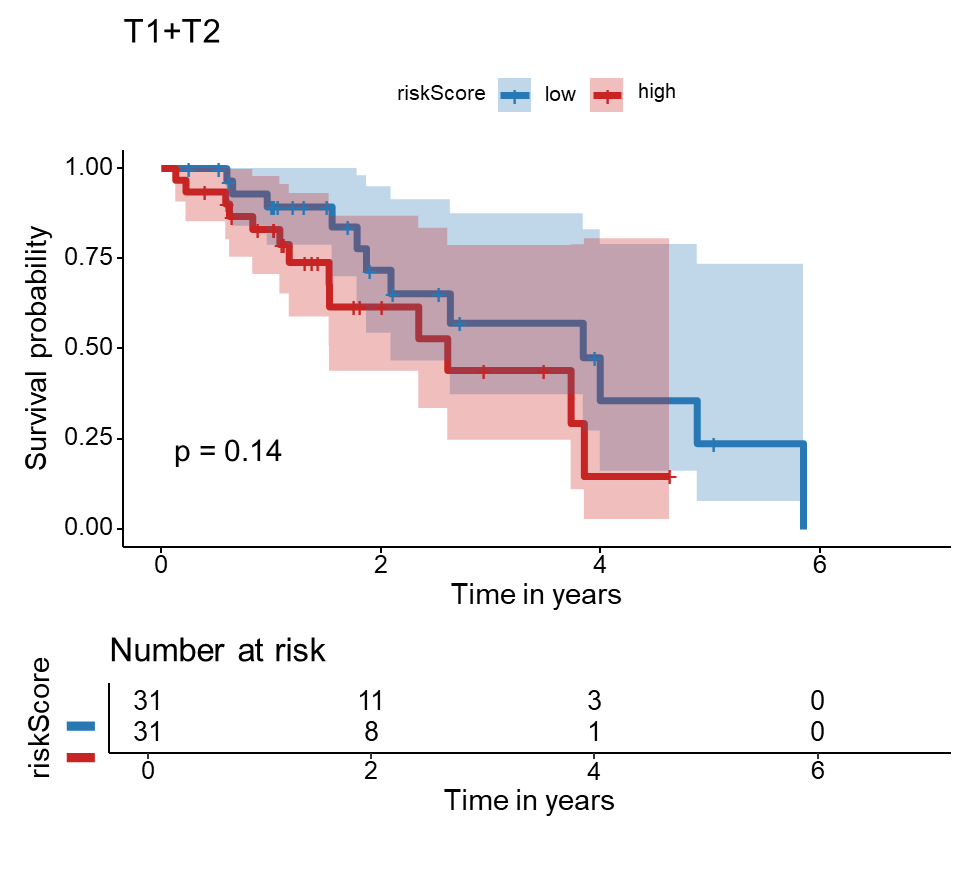

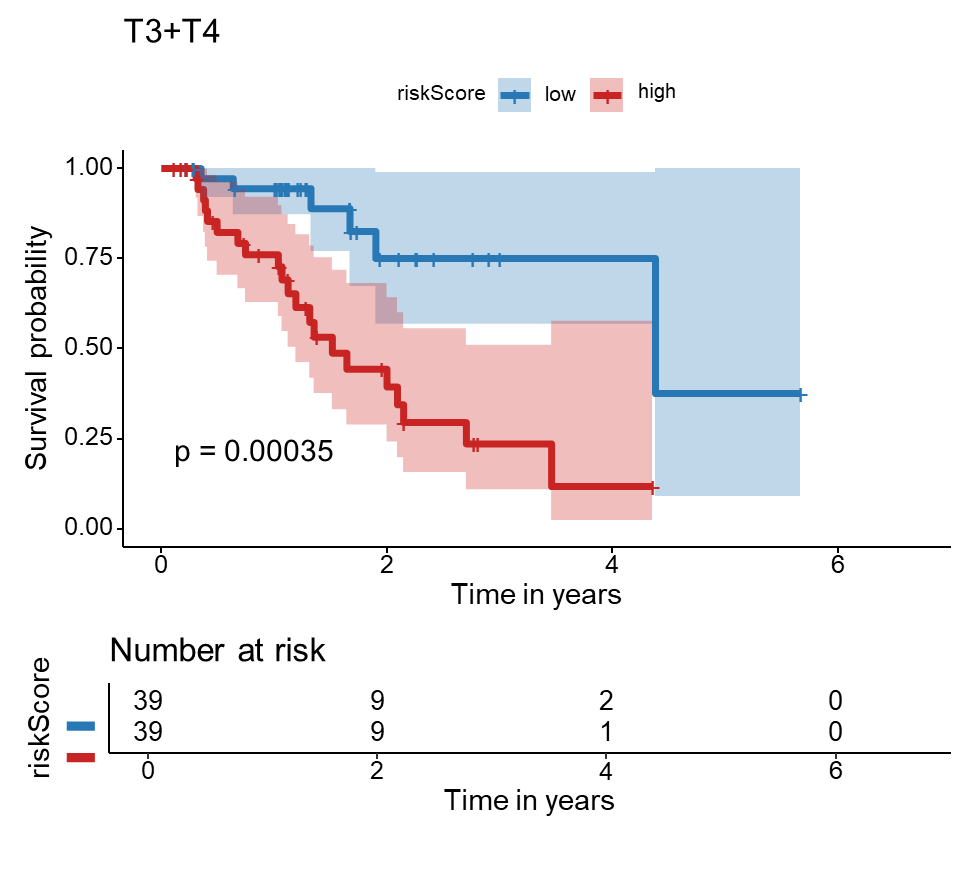

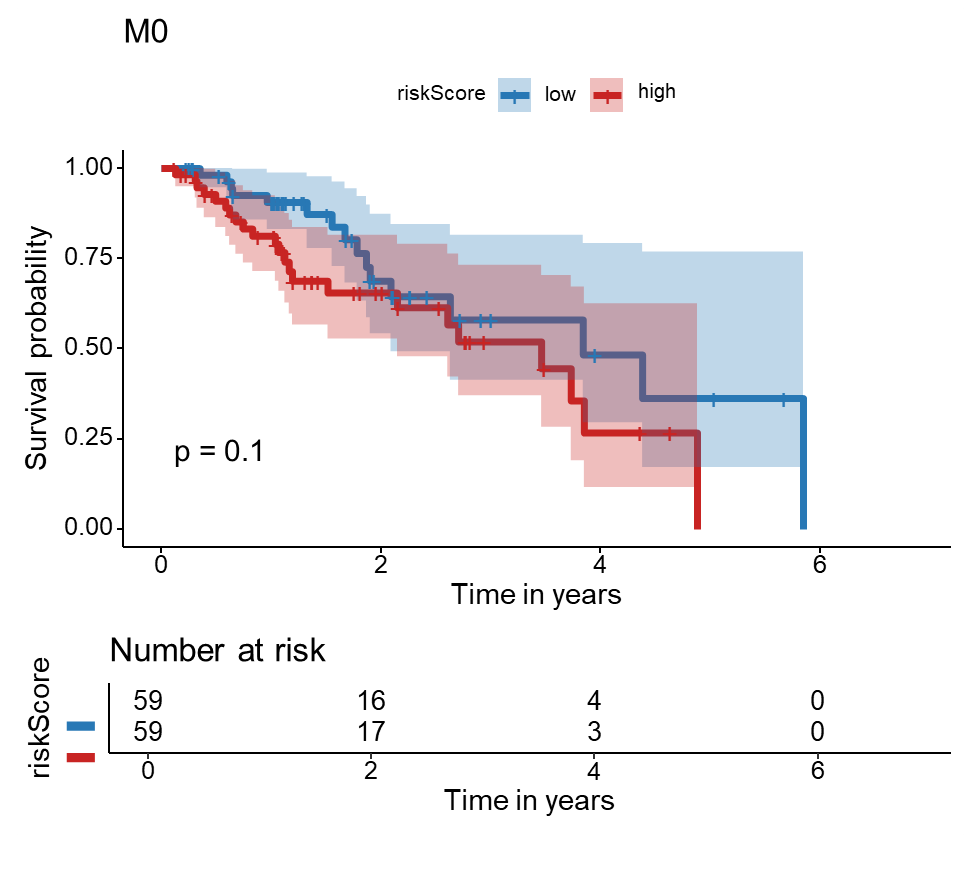

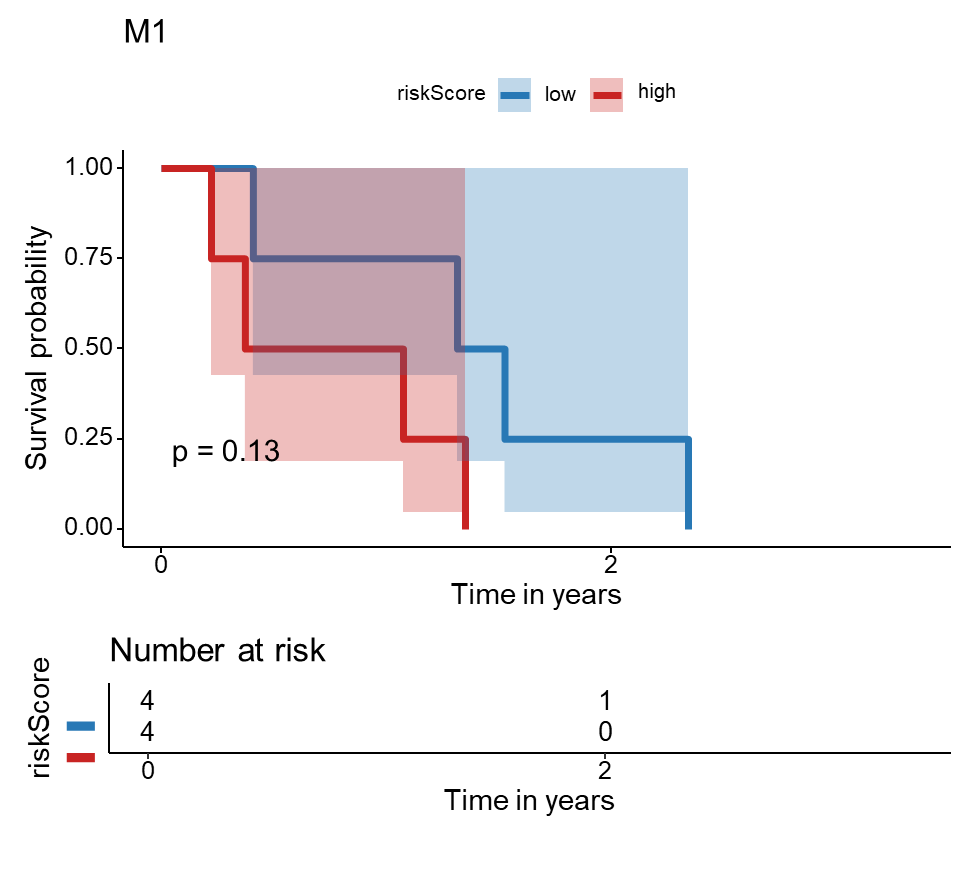

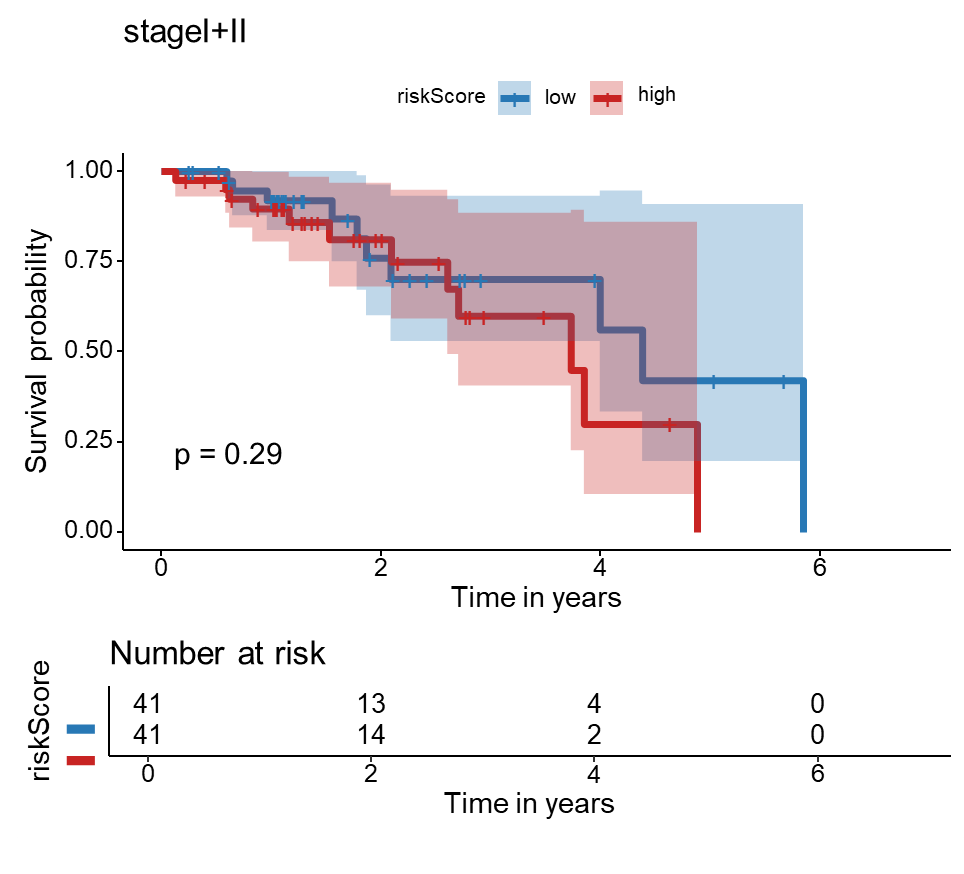

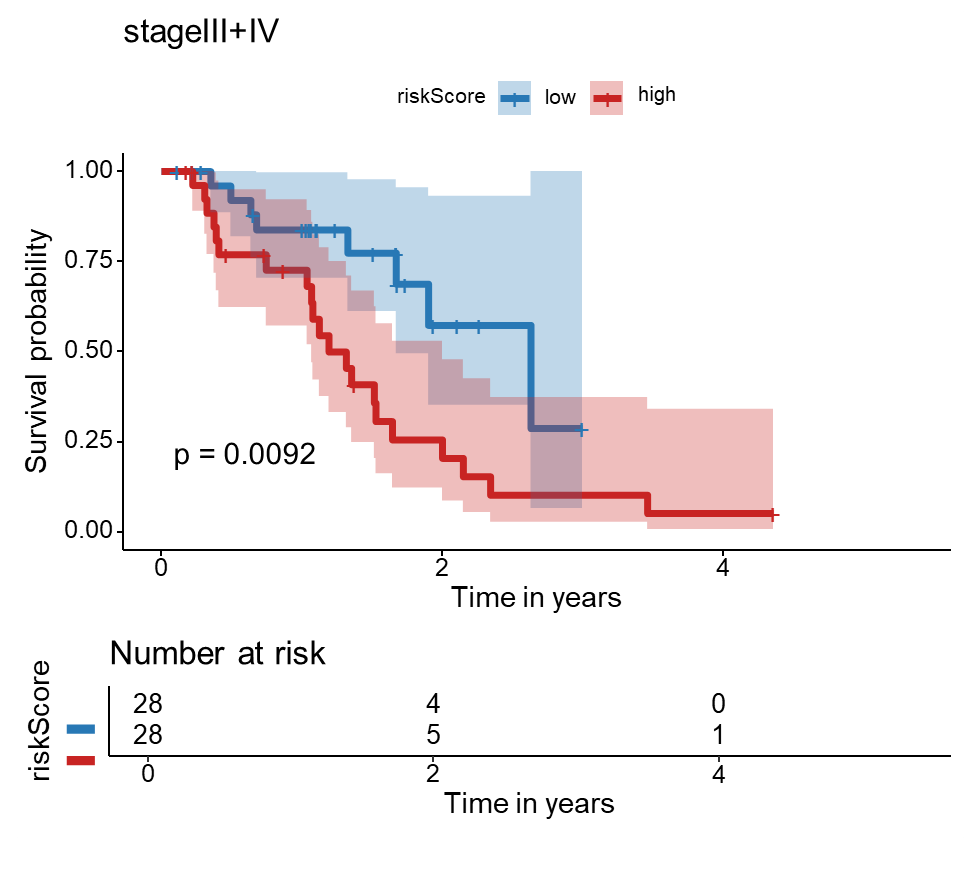

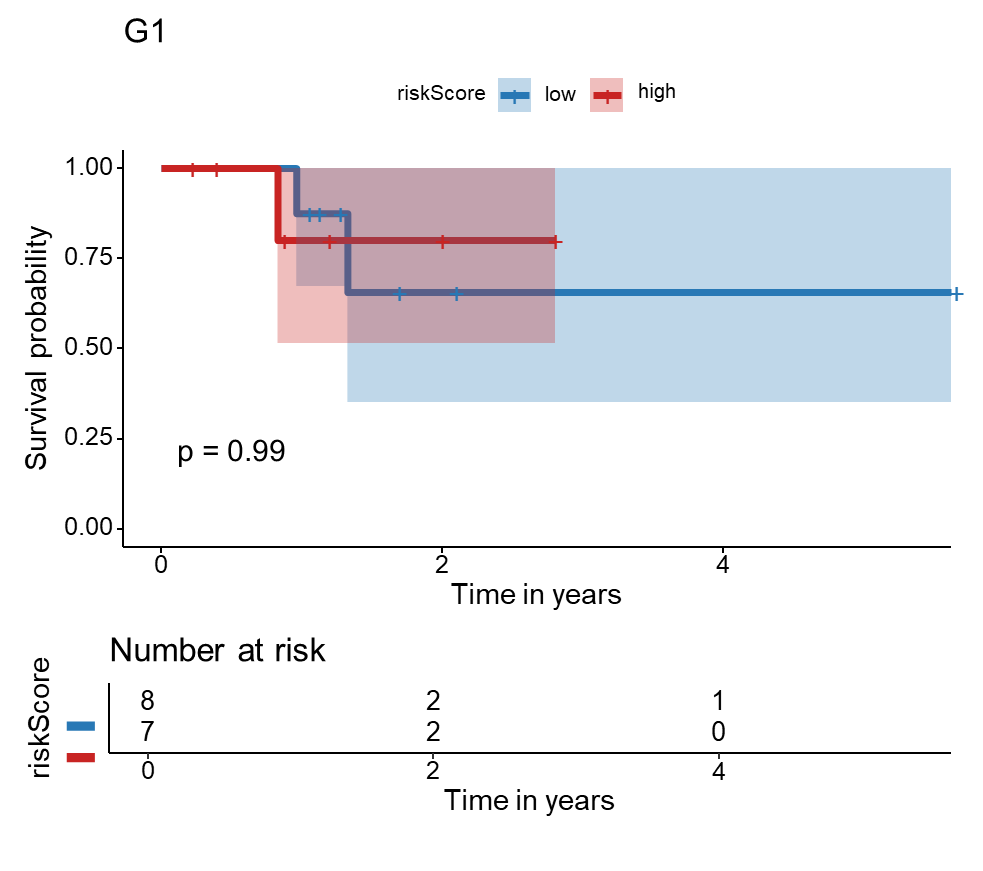

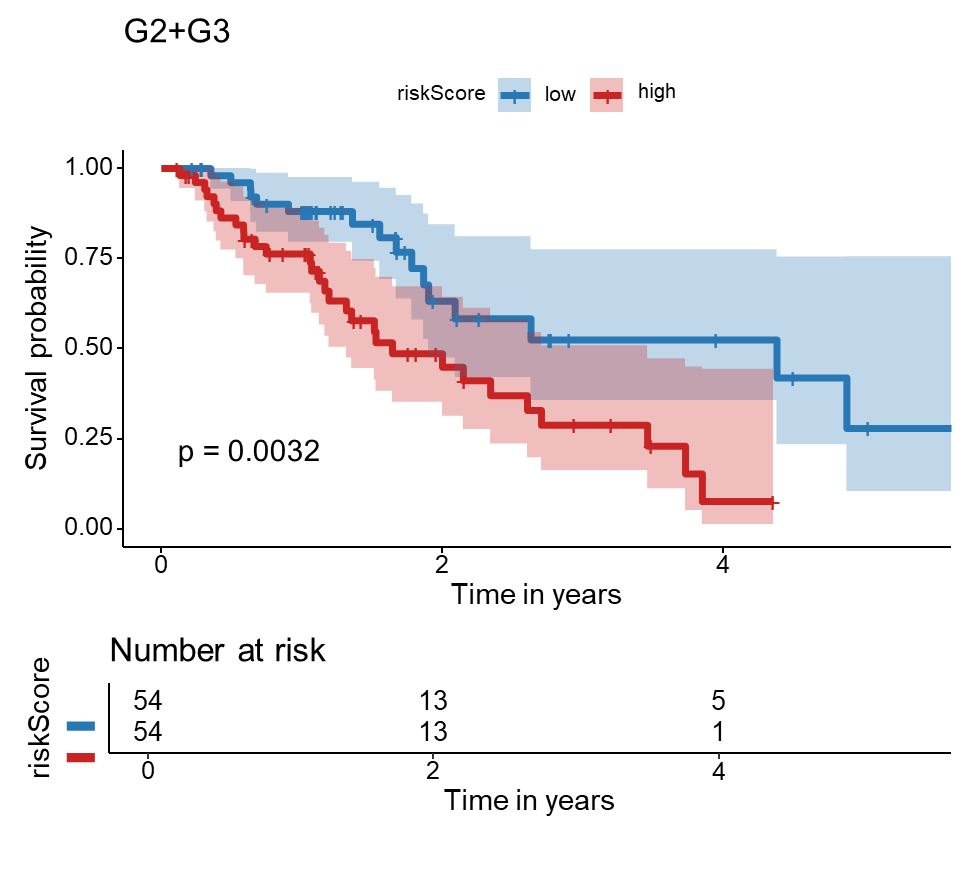

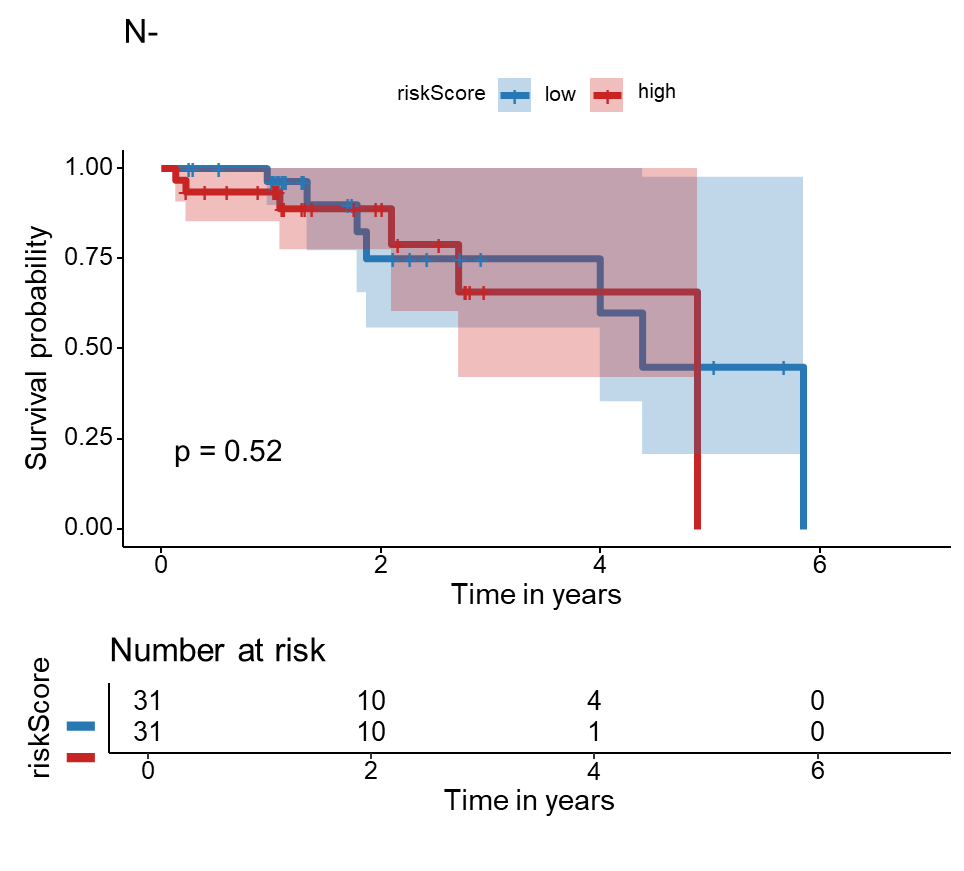

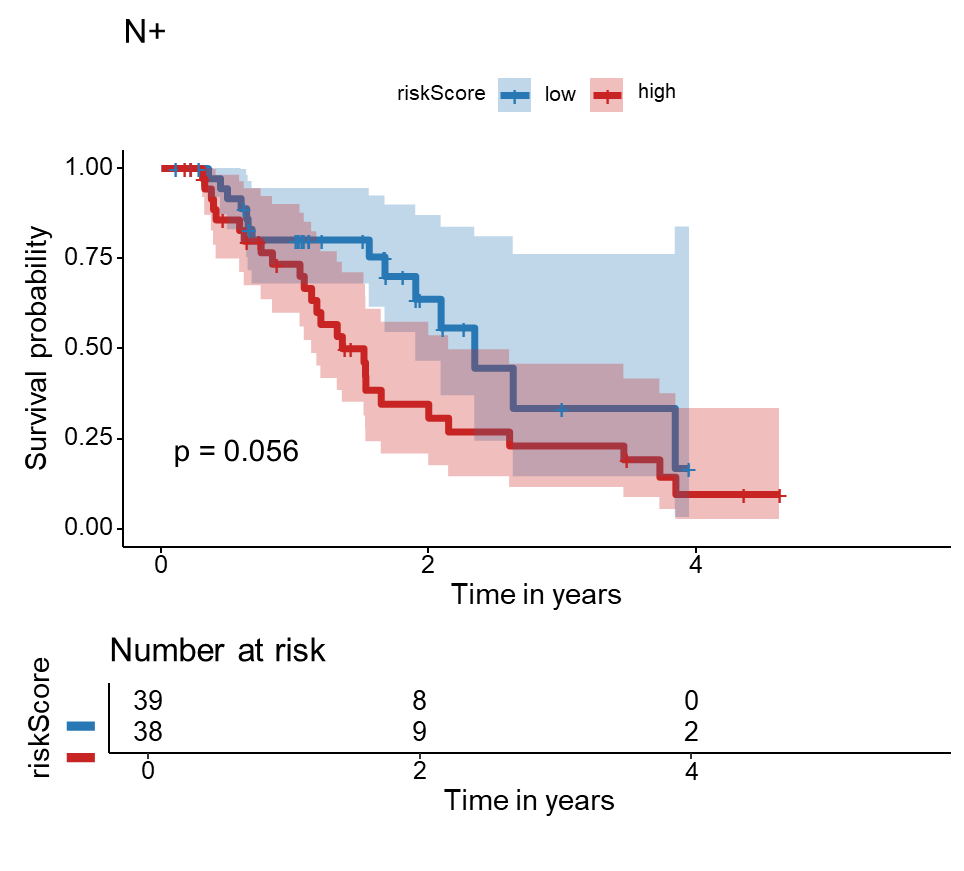


Supplementary Figure 1: Kaplan - Meier (KM) Survival Analysis across Clinical Subgroups Based on Age, Gender, TNM Staging System, Tumor - Stage Categorization, and Tumor - Grade Evaluation
